# Growth differentiation factor 15 protects against the aging‐mediated systemic inflammatory response in humans and mice

**DOI:** 10.1111/acel.13195

**Published:** 2020-07-21

**Authors:** Ji Sun Moon, Ludger J. E. Goeminne, Jung Tae Kim, Jing Wen Tian, Seok‐Hwan Kim, Ha Thi Nga, Seul Gi Kang, Baeki E. Kang, Jin‐Seok Byun, Young‐Sun Lee, Jae‐Han Jeon, Minho Shong, Johan Auwerx, Dongryeol Ryu, Hyon‐Seung Yi

**Affiliations:** ^1^ Research Center for Endocrine and Metabolic Diseases Chungnam National University Hospital Chungnam National University School of Medicine Daejeon Republic of Korea; ^2^ Laboratory of Integrative Systems Physiology École Polytechnique Fédérale de Lausanne (EPFL) Lausanne Switzerland; ^3^ Department of Medical Science Chungnam National University School of Medicine Daejeon Republic of Korea; ^4^ Department of Surgery Chungnam National University School of Medicine Daejeon Republic of Korea; ^5^ Department of Molecular Cell Biology Sungkyunkwan University School of Medicine Suwon Republic of Korea; ^6^ Department of Oral Medicine School of Dentistry Kyungpook National University Daegu Republic of Korea; ^7^ Department of Internal Medicine Korea University College of Medicine Seoul Republic of Korea; ^8^ Department of Internal Medicine School of Medicine Kyungpook National University Daegu Korea; ^9^ Biomedical Institute for Convergence at SKKU (BICS) Sungkyunkwan University Suwon Republic of Korea; ^10^ Samsung Biomedical Research Institute Samsung Medical Center Seoul Republic of Korea

**Keywords:** aging, inflammation, mitochondria, senescence, T cell

## Abstract

Mitochondrial dysfunction is associated with aging‐mediated inflammatory responses, leading to metabolic deterioration, development of insulin resistance, and type 2 diabetes. Growth differentiation factor 15 (GDF15) is an important mitokine generated in response to mitochondrial stress and dysfunction; however, the implications of GDF15 to the aging process are poorly understood in mammals. In this study, we identified a link between mitochondrial stress‐induced GDF15 production and protection from tissue inflammation on aging in humans and mice. We observed an increase in serum levels and hepatic expression of *GDF15* as well as pro‐inflammatory cytokines in elderly subjects. Circulating levels of cell‐free mitochondrial DNA were significantly higher in elderly subjects with elevated serum levels of GDF15. In the BXD mouse reference population, mice with metabolic impairments and shorter survival were found to exhibit higher hepatic *Gdf15* expression. Mendelian randomization links reduced *GDF15* expression in human blood to increased body weight and inflammation. GDF15 deficiency promotes tissue inflammation by increasing the activation of resident immune cells in metabolic organs, such as in the liver and adipose tissues of 20‐month‐old mice. Aging also results in more severe liver injury and hepatic fat deposition in *Gdf15*‐deficient mice. Although GDF15 is not required for Th17 cell differentiation and IL‐17 production in Th17 cells, GDF15 contributes to regulatory T‐cell‐mediated suppression of conventional T‐cell activation and inflammatory cytokines. Taken together, these data reveal that GDF15 is indispensable for attenuating aging‐mediated local and systemic inflammation, thereby maintaining glucose homeostasis and insulin sensitivity in humans and mice.

## Graphical abstract

Aging‐induced GDF15 production is observed in humans and mice, which is positively correlated with systemic inflammation and mitochondrial stress. GDF15 deficiency promotes glucose intolerance as well as hepatic and adipose inflammation in old mice. GDF15 contributes to regulatory T cells‐mediated suppression of conventional T cell activation, but senescent T cells were resistant to regulatory T cells‐mediated suppression compared to conventional T cells.

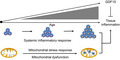

## 1 INTRODUCTION

Aging is a major risk factor for various chronic diseases, including type 2 diabetes, neurodegenerative diseases, and malignancies, which are closely related to systemic subclinical inflammation in the absence of overt infections in the elderly (Ortega Martinez de Victoria et al., 2009; Meda et al., 1995; Multhoff, Molls, & Radons, 2011; Yi et al., 2019). Such systemic inflammatory responses are also termed “metaflammation” or “inflammaging” in humans (Franceschi et al., 2007; Hotamisligil, 2017; Sanada et al., 2018). Given that mitochondrial damage contributes to various senescent processes with a distinct pro‐inflammatory secretory phenotype (Wiley et al., 2016), the fact that the disruption of mitochondrial function is linked to age‐related pathologies is not surprising (Lane, Hilsabeck, & Rea, 2015).

Progressive mitochondrial dysfunction occurs across species during the aging process (Yi, Chang, & Shong, 2018). Oxidative damage to cellular macromolecules, or stress arising from mitochondrial DNA (mtDNA) mutation and increased reactive oxygen species (ROS), is a key hallmark of aging physiology (Yi et al., 2018). Although higher levels of ROS induced by mitochondrial stress are involved in cellular damage and the inflammatory response, they also provide the first line of host defense (Pellegrino et al., 2014). Paradoxically, elevated ROS levels increase the lifespan of worms, flies, and mice through an adaptive response (Yi et al., 2018), termed mitohormesis. The secretion of mitokines during cellular stress is a critical response that may reflect disease severity acting as disease markers. They may also regulate disease progression, which makes them a potential therapeutic target for chronic diseases caused by mitochondrial dysfunction (Yi et al., 2018).

Growth differentiation factor 15 (GDF15) is a well‐known mitokine that is induced by defects in mitochondrial oxidative phosphorylation or by the unfolded protein response (UPR^mt^) pathway in mammals (Chung, Ryu, et al., 2017; Khan et al., 2017). GDF15 production is regulated by the mTORC1 kinase through an integrated mitochondrial stress response in patients with mitochondrial myopathy (Khan et al., 2017). Plasma GDF15 levels increase during metabolic stress‐mediated tissue inflammation, including in insulin resistance and type 2 diabetes (Kempf et al., 2012; Yi, 2019). Skeletal muscle‐specific UPR^mt^ is also related to the promotion of lipolysis and fatty acid oxidation in adipose tissues by GDF15 production, thereby protecting the organism against high fat, diet‐induced obesity, and insulin resistance (Chung, Ryu, et al., 2017). Additionally, GDF15 improves alcohol‐ or chemically induced chronic liver injury by suppressing the infiltration of neutrophils, monocytes, and activated T cells in the liver (Chung, Kim, et al., 2017). Although the fibroblast growth factor 21 (FGF21) maintains peripheral T‐cell homeostasis by attenuating thymic immune senescence with age (Youm, Horvath, Mangelsdorf, Kliewer, & Dixit, 2016), the immunometabolic role of GDF15 in the aging process is incompletely understood.

In this study, we provide evidence that GDF15 exerts a protective effect on tissue inflammation in metabolic organs, such as the liver and adipose tissues, in humans and mice. Through complementary human and animal experiments supported by the reanalysis of large‐scale human transcript datasets, we demonstrate that GDF15 is required for the prevention of aging‐induced development of metabolic diseases by regulating tissue and systemic inflammation. Taken together, the immune regulatory role of GDF15 reveals the dynamic interplay between the metabolic and immune systems and contributes to delays in aging‐induced systemic inflammation.

## 2 RESULTS

### 2.1 Elderly subjects exhibit higher levels of serum GDF15 and hepatic GDF15 expression

**Figure 1:**
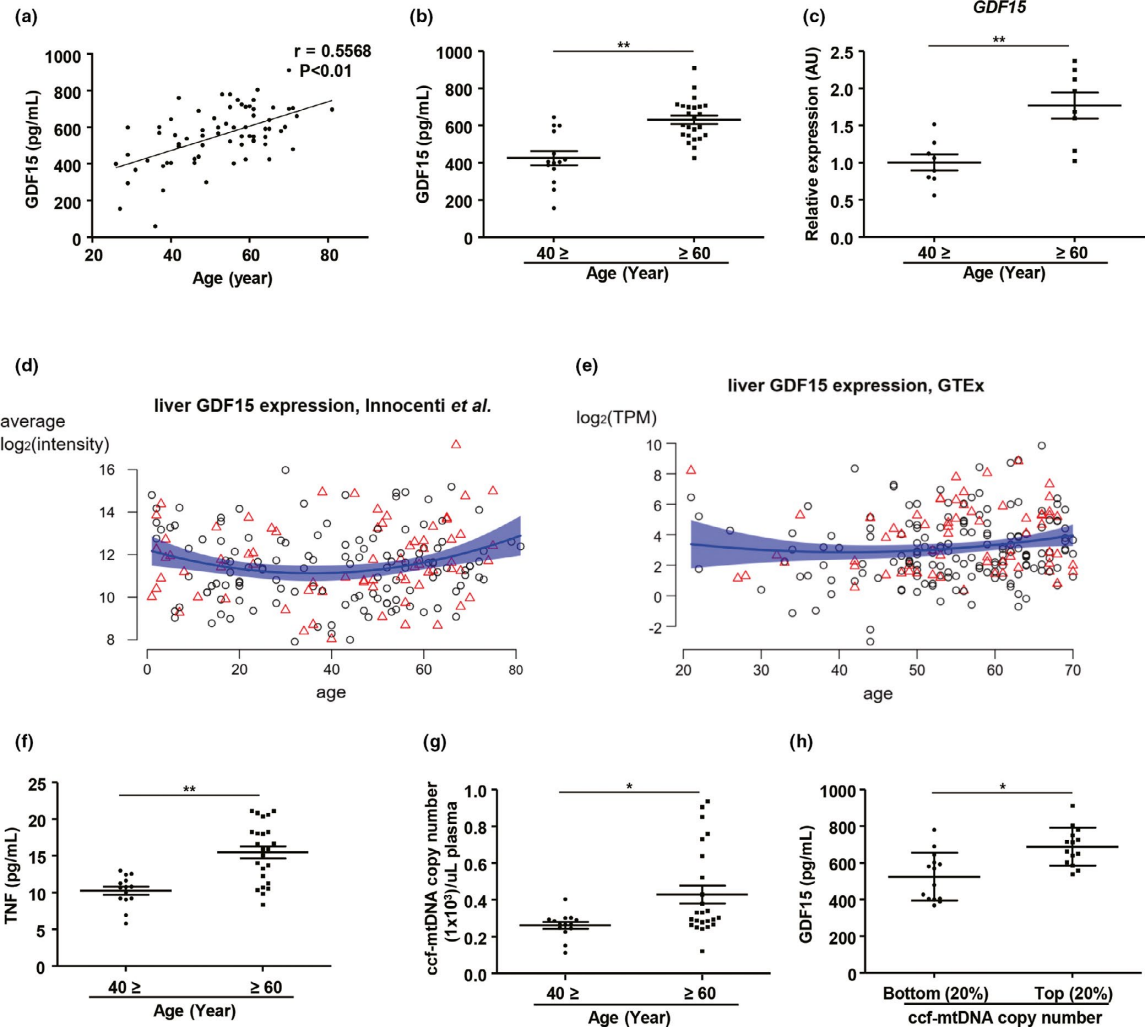
GDF15 correlates positively with aging‐induced systemic inflammation in humans. (a) Correlation analysis of serum GDF15 levels in human subjects. (b) Serum levels of GDF15 in young (**≤**40; n = 14) and elderly (≥60; n = 24) subjects. (c) Hepatic expression of *GDF15* in young (**≤**40; n = 8) and elderly (≥60; n = 8) subjects. (d,e) The effect of age on hepatic *GDF15* expression in (d) a microarray dataset showing patient‐averaged hepatic log_2_‐transformed *GDF15* intensities for 202 patients (Innocenti et al., 2011), and (e) a GTEx RNA‐Seq dataset with log_2_‐transformed *GDF15* expression in transcripts per million (TPM) for 226 liver biopsies. Men are denoted as black circles, women as red triangles. The blue trend lines are obtained by fitting regression models with linear and quadratic age effects to the data. The transparent blue bands denote the 95% confidence intervals corresponding to these models. (f) Serum levels of TNF in young (**≤**40; n = 14) and elderly (≥60; n = 24) subjects. (g) Quantitation of mtDNA levels in ccf‐DNA from plasma in study participants. (h) Serum levels of GDF15 in subjects with the 20% lowest (bottom; n = 14; mean age, 46.4 years old) or 20% highest (top; n = 14; mean age, 65.5 years old) plasma levels of ccf‐mtDNA copy numbers. Data are expressed as mean ± SEM. *p < 0.05, **p < 0.01 ((a): simple linear regression, (b,c), (f–h): two‐tailed t‐tests)

**Figure 2:**
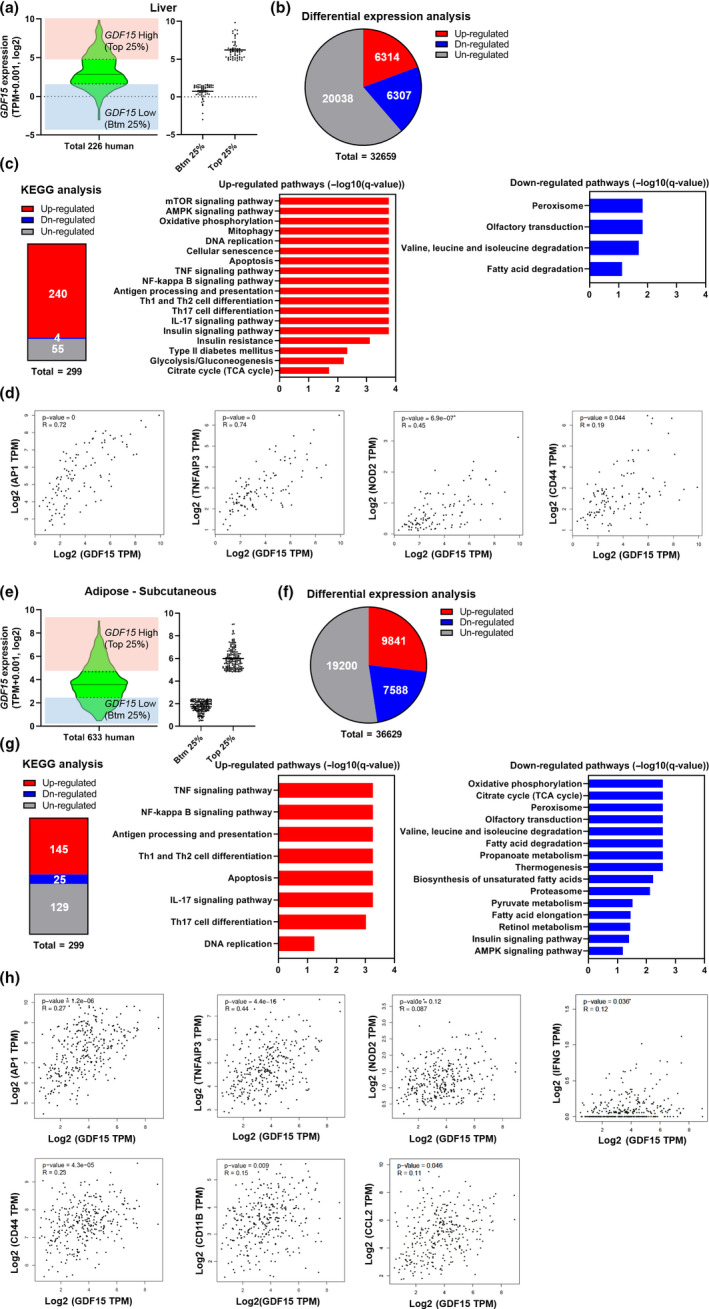
Analysis of related pathways regulated by *GDF15* expression in liver and adipose tissue in the GTEx database. (a) Distribution of 226 hepatic *GDF15* expression levels (log_2_(TPM + 0.001)) for human subjects in GTEx. The red and blue boxes represent the top 25% (n = 57) and the bottom 25% (n = 57) of the group according to *GDF15* expression levels, respectively. (b) Number of DEGs between the top 25% and the bottom 25% according to *GDF15* levels. The red, blue, and gray represent the number of up‐regulated, down‐regulated, and un‐regulated genes, respectively. (c) KEGG pathway analysis of the DEA results. The red, blue, and gray boxes indicate up‐regulated, down‐regulated, and un‐regulated pathways, respectively. Bar plots representing the up‐regulated (red) and down‐regulated (blue) pathways for significantly enriched pathways. The pathways shown in these bar plots were selected from the significant pathways (FDR <0.1) in the KEGG analysis. (d) Hepatic *GDF15* expression correlates positively with *AP1*,* TNFAIP3*,* NOD2*, and *CD44* gene expression. The correlation analysis was conducted by GEPIA2 in the GTEx liver dataset (R: Pearson's correlation coefficient). (e) Distribution of 633 subcutaneous adipose tissue *GDF15* expression levels (log_2_(TPM + 0.001)) for human subjects in GTEx. The red and blue boxes represent the top 25% (n = 158) and bottom 25% (n = 158) groups according to *GDF15* levels, respectively. (f) Number of DEGs between the top 25% and bottom 25% groups according to *GDF15* levels. The red, blue, and gray boxes represent the number of up‐regulated, down‐regulated, and un‐regulated genes, respectively. (g) KEGG pathway analysis of the DEA results. The red, blue, and gray boxes indicate up‐regulated, down‐regulated, and un‐regulated pathways, respectively. Bar plots represent the up‐regulated (red) and down‐regulated (blue) pathways for significantly enriched pathways. The pathways shown in these bar plots were selected from the significant pathways (FDR <0.1) in the KEGG analysis. (h) Adipose tissue *GDF15* expression correlates positively with *AP1*,* TNFAIP3*,* NOD2*,* IFNG*,* CD44*,* CD11B*, and *CCL2* gene expression. The correlation analysis was conducted by GEPIA2 in the GTEx subcutaneous adipose tissue dataset (R: Pearson's correlation coefficient)

In an experiment on 12 male C57BL/6 WT mice, we noticed that serum GDF15 levels were elevated in old (20‐month‐old) mice compared to young (8‐week‐old) mice (Figure S1a). Subsequently, we recruited 70 participants of which the demographics and baseline characteristics are summarized in Table S1. Again, we observed a significant positive association between the age of the study subjects and serum levels of GDF15 (Figure 1a). Elderly subjects (≥60 years) also exhibited significantly higher levels of serum GDF15, compared with younger subjects (≤40 years) (Figure 1b). We further noticed that hepatic *Gdf15* expression was higher in old mice (20‐month‐old) compared to young mice (8‐week‐old) (Figure S1b). Likewise, hepatic *GDF15* expression was remarkably increased in elderly subjects compared with young people (Figure 1c). We confirmed this age‐related increase in hepatic GDF15 expression in two independent large human transcript datasets: (1) a liver microarray dataset (Innocenti et al., 2011) (Figure 1d) and (2) the RNA‐Seq data of the human Genotype‐Tissue Expression (GTEx) project (Consortium, 2015) (Figure 1e). In both datasets, GDF15 expression decreases in very young subjects (up to 30 years old), remains constant between 30 and 50 years of age, and then increases again after 50 years old. These non‐linear age effects are significant in both the microarray dataset (limma analysis, *p* = 4 × 10^−5^) and the GTEx RNA‐Seq dataset (edgeR‐zingeR analysis, *p* = 0.04). The former would even remain significant in a genome‐wide screen (q‐value =0.001). Average *GDF15* expression is 65% higher in 60‐ to 81‐year‐old subjects as compared to 20‐ to 40‐year‐old subjects (corrected for gender and ancestry, *p* = 0.06) (Innocenti et al., 2011). *Gdf15* is also highly expressed in murine livers compared to other tissues (Figure S1c). If we equate 6‐months‐old mice to 30‐year‐old humans and 14‐month‐old mice to 50‐year‐old humans (Fox, 2007), this trend can also be observed in C57BL/6 JN mice (Figure S1d) (Tabula Muris et al., 2018). The lower *Gdf15* expression in very old mice (27 months old) might be due to survival bias as only ~50% of mice reach this age.

### 2.2 GDF15 is linked to inflammation and mitochondrial stress

We also found that elderly subjects exhibited higher levels of serum TNF and increased hepatic TNF expression (Figure 1f and Figure S1e). The subjects with a higher level (top 25%) of serum GDF15 exhibited significantly elevated hepatic TNF expression compared with those with lower levels (bottom 25%) of serum GDF15 (Figure S1f). Old subjects also showed a reduction in the frequency of naïve (CD45RA^+^CD45RO^−^) CD4^+^ and CD8^+^ T cells and an increase in the frequency of memory (CD45RA^−^CD45RO^+^) CD4^+^ and CD8^+^ T cells in the peripheral blood (Figure S1g–i). Moreover, the absolute numbers of naïve CD4^+^ and CD8+ T cells were reduced in older subjects, but the number of memory CD8^+^ T cells was increased (Figure S1j). In addition, the population of memory CD8^+^ T cells showed a positive correlation with serum levels of GDF15 (Figure S1k). The production of granzyme B in senescent CD4^+^ and CD8^+^ T cells was also higher in elderly subjects, compared with young subjects (Figure S1l–o).

Mitochondrial damage is closely linked with the systemic inflammatory response in human diseases, and circulating mitochondrial DNA is also associated with inflammation during aging (Picca et al., 2018). GDF15 is a well‐known mitokine that is secreted during mitochondrial stress and damage. Thus, we also measured mitochondria DNA (mtDNA) levels by quantitating the copy number of mitochondria DNA in circulating‐cell‐free (ccf) DNA in the plasma of young and elderly subjects. Although there is a high variability in circulating mtDNA in the elderly population which may be explained by individual differences in mitochondrial dysfunction, the mtDNA levels were significantly higher in elderly subjects compared with the younger controls (Figure 1g). Subjects with higher levels (top 20%) of ccf‐mtDNA copy number in plasma exhibited significantly higher levels of serum GDF15 compared with those with lower levels (bottom 20%) (Figure 1h). In addition, GDF15 levels were positively correlated with ccf‐mtDNA copy number in plasma (Figure S2a). We also found that tissue‐specific deficiency of CR6‐interacting factor‐1 (Crif1)‐induced dysfunction of mitochondrial oxidative phosphorylation exhibited an increase in serum levels of GDF15 in mice at 8 weeks of age compared with controls (Figure S2b) (Choi et al., 2020; Chung, Ryu, et al., 2017). Collectively, our data show that increases in both hepatic *GDF15* expression and serum levels of GDF15 are associated with aging‐related inflammation and mitochondrial damage.

### 2.3 Analysis of transcriptome datasets from the Genotype‐Tissue Expression (GTEx) project

To further investigate the relationship between *GDF15* and inflammatory response at the transcriptome level, we utilized GTEx RNA‐Seq data from the liver, adipose tissue, and skeletal muscle to observe whether *GDF15* expression is associated with systemic inflammation in humans. Differential expression gene analysis (DEA) was performed by dividing the data into two groups (top 25% and bottom 25% group) based on *GDF15* expression levels. First, DEA was performed in the liver (Figure 2a). The –log10(q‐value) for *GDF15* was equal to 191.7, confirming that each group was well‐differentiated by the expression of *GDF15* (Figure S3a). The DEA results indicated that 6,314 up‐regulated and 6307 down‐regulated genes differed between the *GDF15* top 25% group and the bottom 25% group (Figure 2b). Next, pathway analysis using the Kyoto Encyclopedia of Genes and Genomes (KEGG) was performed to define pathways that differ between groups in terms of *GDF15* expression. Of the total 299 available pathways, the top 25% group with the highest *GDF15* levels exhibited 240 up‐regulated pathways and 4 down‐regulated pathways compared with the bottom 25% group. We found that mTOR signaling pathway and mitochondria‐related pathways were up‐regulated in the *GDF15* top 25% group compared to the *GDF15* bottom 25% group. We also observed an increase in inflammation‐related pathways, including the TNF signaling and IL‐17 signaling pathways. On the other hand, the pathways related valine, leucine and isoleucine degradation, and fatty acid degradation were significantly down‐regulated (Figure 2c). A correlation analysis on the GTEx liver expression data revealed that the expression levels of *AP1*, *TNFAIP3*, *NOD2*, and *CD44*, which play important roles in inflammation including TNF signaling, showed significant positive correlation with *GDF15* expression (Figure 2d).

The role of GDF15 in subcutaneous adipose tissue was analyzed next. As in the liver, groups were divided into top 25% and bottom 25% groups, according to *GDF15* expression levels (Figure 2e). The q‐value for *GDF15* was approximately 0, indicating that each group was well‐divided in terms of *GDF15* expression (Figure S3b). For the DEA results, the top 25% group contained 9841 up‐regulated genes and 7588 down‐regulated genes, compared with the bottom 25% group (Figure 2f). KEGG analysis showed that inflammation‐related pathways were up‐regulated and mitochondria‐related pathways were down‐regulated, similar to what we observed in the liver (Figure 2g). Correlation analyses showed that, in addition to the inflammation‐related genes (*AP1*,* TNFAIP3*,* NOD2*, and *CD44*) observed in the liver, genes such as *IFNG*, *CD11B*, and *CCL2* also correlated positively with *GDF15* mRNA expression levels (Figure 2h). Similar data were observed in skeletal muscle (Figure S4a–d).

### 2.4 Role of GDF15 on longevity and metabolic phenotypes in mouse populations

**Figure 3:**
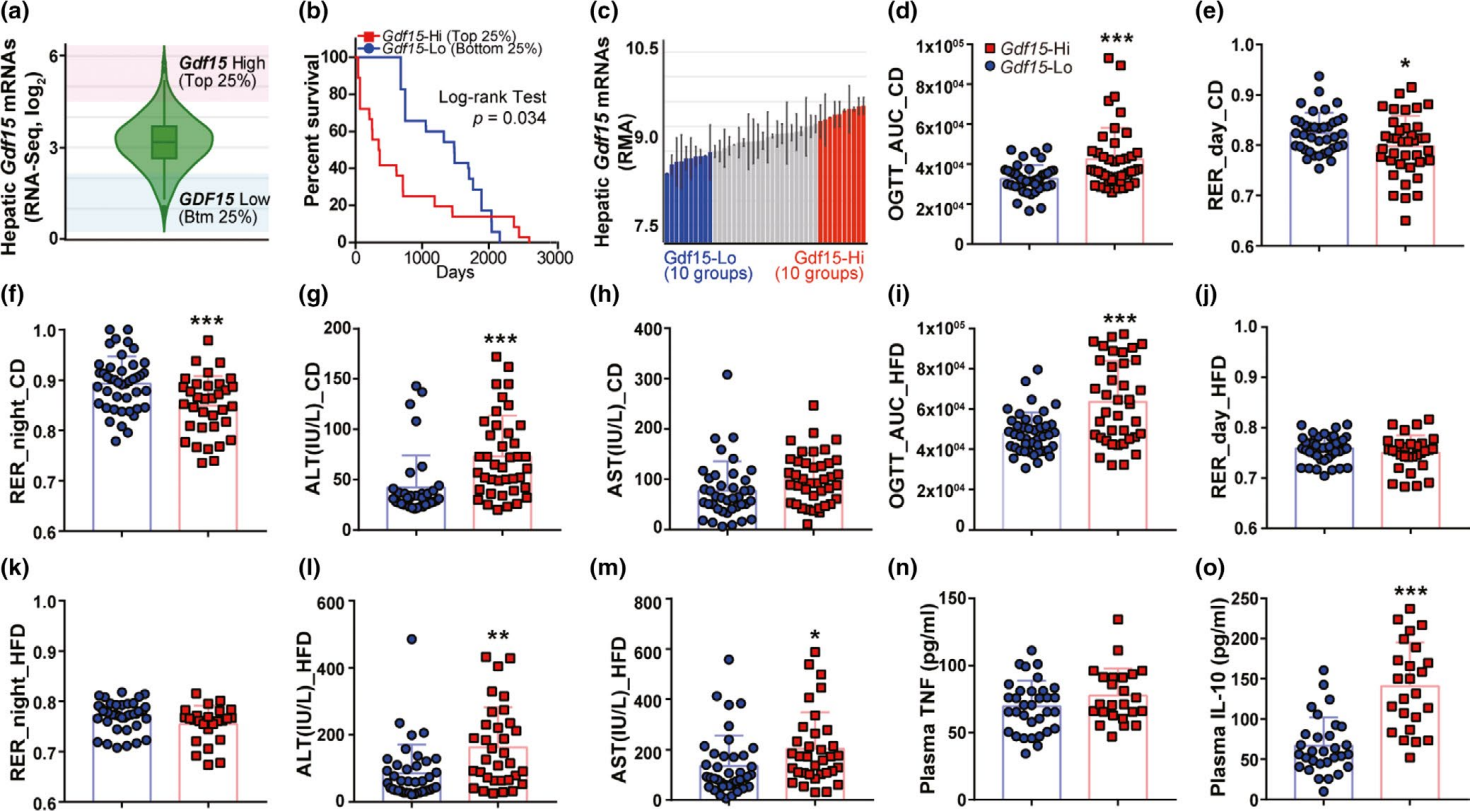
Impact of *Gdf15* on metabolic phenotypes and survival in BXD mouse reference populations. (a) Violin plot visualizing the distribution of 241 BXD mice by hepatic *Gdf15* expression. The red and blue boxes represent the top and bottom 25% of BXD populations, respectively. (b) Kaplan–Meier plot showing the survival curves for the top (n = 37) and bottom (n = 36) 25% of mice corresponding to the red and blue squares, regardless of their BXD line (log‐rank (Mantel–Cox) test *p* = 0.039; Gehan–Breslow–Wilcoxon test *p* < 0.0001, hazard ratio = 0.5644). (c) The mean expression of hepatic *Gdf15* in each BXD strain at 29 weeks of age. The *Gdf15*‐low (blue) or *Gdf15*‐Hi (red) group consisted of 10 BXD lines each with, respectively, the lowest and highest hepatic *Gdf15* expression levels. (d–o) Metabolism and inflammation‐related phenotypes of *Gdf15*‐low (blue circle) and *Gdf15*‐Hi (red square) groups (n = 3 to 5 mice per BXD line). The area under curve for the oral glucose tolerance test (OGTT AUC) (d), the respiratory exchange ratio (RER) during day and night (e,f), alanine aminotransferase (ALT), and aspartate aminotransferase (AST) (g, h) were obtained from the *Gdf15*‐low (blue) or *Gdf15*‐Hi (red) groups fed a normal chow diet at 29 weeks of age. The OGTT AUC (i), RER_day (j), RER_night (k), AST (l), ALT (m), plasma TNF (n), and plasma IL‐10 (o) were from the *Gdf15*‐low (blue) or *Gdf15*‐Hi (red) groups under a high‐fat diet at 29 weeks of age. Values (d–o) are mean ± SEM. *p < 0.05, **p < 0.01, ***p < 0.001 ((d–o): two‐tailed t tests)

To further explore the link between GDF15 and the metabolic features of aging, we analyzed whether GDF15 impacts lifespan and metabolic phenotypes in the BXD mouse genetic reference population, which is composed of ~160 genetically different mouse strains (Andreux et al., 2012). First, we analyzed the overall impact of hepatic *Gdf15* expression on murine lifespan without considering their strain (Figure 3a, b). BXD mice with high expression (top 25%) of *Gdf15* transcripts in the liver lived significantly shorter than mice with low expression levels (bottom 25%) (Figure 3a,b). Higher *Gdf15* expression in the liver is tightly associated with gene sets involved in mitochondrial stress and quality control (Figure S5a,b). To identify the *Gdf15*‐expression‐associated metabolic phenotypes, we also analyzed the major phenotypes of the individual BXD mice with higher (top 25%; *Gdf15*‐Hi) or lower expression (bottom 25%; *Gdf15*‐Lo) of hepatic *Gdf15* transcripts (Figure 3c). The *Gdf15*‐Hi BXD strains placed on a normal chow diet exhibited glucose intolerance (Figure 3d) and a lower respiratory exchange ratio (RER) compared with the *Gdf15*‐Lo strains (Figure 3e,f). Although we found no differences in food intake between chow‐fed *Gdf15*‐Lo and *Gdf15*‐Hi animals, body weight (Figure S6a,b) and fat mass were significantly higher in the latter, whereas the lean mass was not altered (Figure S6c,d). The chow‐fed *Gdf15*‐Hi group also had heavier liver masses (Figure S5e) and higher serum levels of liver injury markers (Figure 3g,h) compared to the chow‐fed *Gdf15*‐Lo group.

Then, we assessed the metabolic parameters in the same BXD strains but now fed with a high fat diet (HFD) for 21 weeks. Consistent with the data from the chow‐fed mice, the *Gdf15*‐Hi strain fed with a HFD developed more severe glucose intolerance (Figure 3i) without any changes in food intake (Figure S6f) compared with the *Gdf15*‐Lo strain, but the *Gdf15*‐Hi group also showed a similar RER compared with the *Gdf15*‐Lo group (Figure 3j,k). The *Gdf15*‐Hi group exhibited more severe hepatocellular injury (Figure 3l,m), although food intake, body weight, body composition, and liver mass were not clearly altered (Figure S6f‐j). Moreover, the *Gdf15*‐Hi BXD mice were cold intolerant compared with *Gdf15*‐Lo mice, both when fed a chow or a HFD (Figure S6k,l). In the *Gdf15*‐Hi group, the plasma levels of TNF, interleukin 10 (IL‐10), and monocyte chemoattractant protein 1 (MCP1) were increased, but only differences in IL‐10 levels passed the significance threshold of 5% (Figure 3n,o and Figure S6m). These data demonstrate that mice with higher hepatic *Gdf15* expression exhibit metabolic impairment and tissue inflammation.

### 2.5 Mendelian randomization study with human data

**Figure 4:**
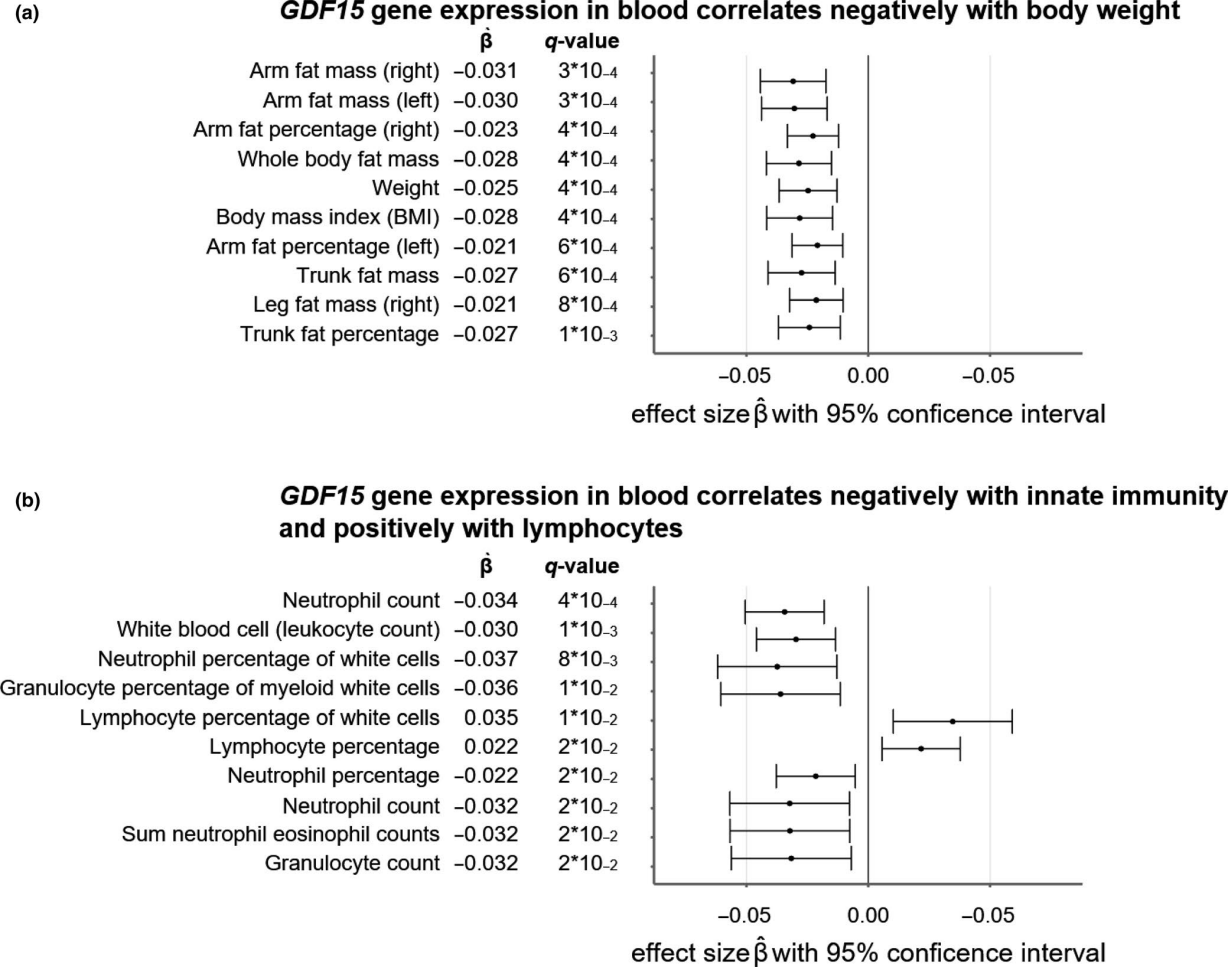
Mendelian randomization of GTEx liver *GDF15* expression in whole blood through SNP rs7226 on 33 obesity‐related outcomes and 27 outcomes related to blood leukocyte concentrations. Wald ratio tests were used to test for statistical significance. q‐values were calculated with the Benjamini–Hochberg multiple testing FDR procedure. All estimated effect sizes $\hat{\beta }$ should be interpreted as average increases (for positive effect sizes) or decreases (for negative effect sizes) in the outcomes per unit increase in normalized effect size (NES) *GDF15* expression. Masses and weights are expressed in kg; BMI is expressed in kg/m^2^. Whiskers denote 95% confidence intervals. (a) Top 10 most significantly changing obesity‐related traits; (b) top 10 most significantly changing immunity‐related traits

We then performed a Mendelian randomization through the single nucleotide polymorphism (SNP) rs7226, which is significantly associated with *GDF15* expression in whole blood in GTEx and has been linked to GDF15 blood concentration before (Jiang et al., 2018). We assessed 33 obesity‐related traits and 27 traits related to concentration of leukocytes in the blood obtained from a variety of publicly available human GWAS datasets (Table S2). Increased *GDF15* expression was significantly associated with reduced obesity and fat mass for 24 traits at 5% false discovery rate (FDR) (Table S2, top 10 shown in Figure 4a). This is unsurprising, as GDF15 is known to prevent obesity (Chung, Ryu, et al., 2017; Tsai, Lin, Brown, Salis, & Breit, 2016). However, increased *GDF15* expression through rs7226 also appears to be linked to a decreased concentration of total leukocytes, innate immune cells, and myeloid white cells (9 significant traits at 5% FDR) and an increased concentration of lymphocytes and monocytes in the blood (3 significant traits at 5% FDR) (Table S2, top 10 shown in Figure 4b).

### 2.6 GDF15 depletion produces hepatic and adipose inflammatory responses during the aging process

**Figure 5:**
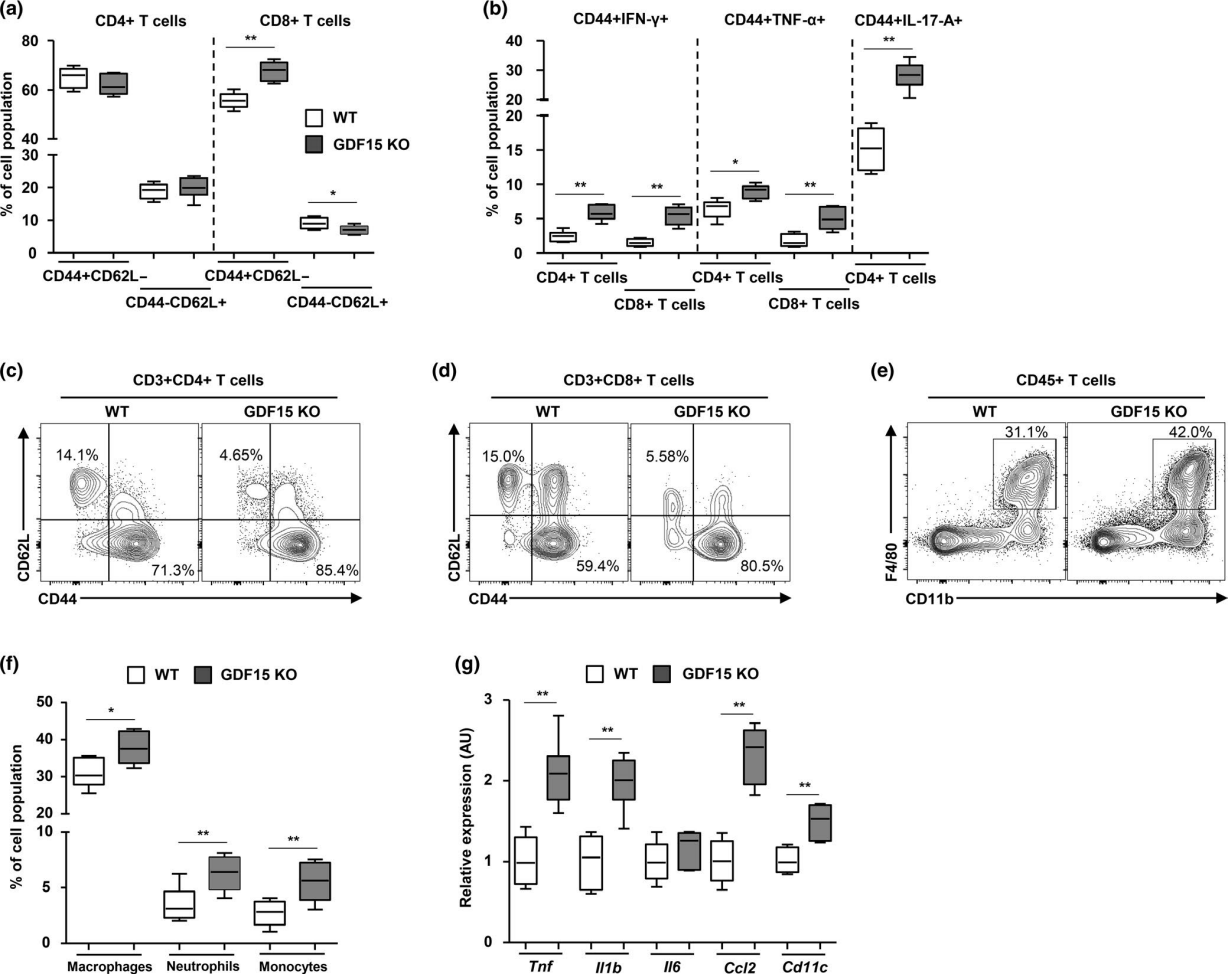
GDF15‐mediated regulation of the inflammatory response in the liver and gonadal adipose tissues during aging. (a) Population size and frequency of CD44^+^CD62L^−^ and CD44^−^CD62L^−^ in CD4^+^, and CD8^+^ T cells in liver tissues of 20‐month‐old WT (n = 6) or *Gdf15* KO (n = 6) mice. (b) IFN‐γ, TNF‐α, or IL‐17A producing CD4^+^ and CD8^+^ T cells in liver tissues of 20‐month‐old WT (n = 6) or *Gdf15* KO (n = 6) mice. (c,d) Representative flow cytometry plots of CD44^+^CD62L^−^ and CD44^−^CD62L^−^ in CD4^+^, and CD8^+^ T cells in liver tissues of 20‐month‐old WT (n = 6) or *Gdf15* KO (n = 6) mice. (e) Percentage of CD11b and F4/80‐positive cells within the livers of 20‐month‐old WT and Gdf15 KO mice. (f) Frequencies of infiltrating macrophages, monocytes, and neutrophils in liver tissues of WT (n = 6) or *Gdf15* KO (n = 6) mice. (g) Transcript levels of pro‐inflammatory cytokines in liver tissues of WT (n = 6) or *Gdf15* KO (n = 6) mice. Data are expressed as mean ± SEM. *p < 0.05, **p < 0.01 ((a,b,f,g): two‐tailed t tests)

Based on the link of GDF15 with aging, metabolism, and inflammation observed in mouse and humans and the abnormalities in metabolic tissues (liver, muscle, adipose tissue) in the BXD mouse reference population, we investigated the population and function of immune cells in the liver of 20‐month‐old WT and *Gdf15* knockout (KO) mice (Figure S7). We found no remarkable difference in the absolute number of liver mononuclear cells between the 20‐month‐old WT and *Gdf15* KO mice (Figure S8a). In the subset analysis of liver mononuclear cells (Figure S7b), the population of natural killer T cells and mature CD3+ T cells did not exhibit changes in both the 20‐month‐old WT or *Gdf15* KO mice, but the number of natural killer cells was significantly decreased in the liver of *Gdf15* KO old mice (Figure S8a–c). 20‐month‐old *Gdf15* KO mice exhibited a higher population of CD8^+^ T cells and a lower population of CD4^+^ T cells in the liver compared with 20‐month‐old WT mice (Figure S8b–d). Although the frequency of naïve and memory CD4^+^ T cells was not different between the WT and *Gdf15* KO old mice, the population of memory CD8^+^ T cells and naïve CD8^+^ T cells was, respectively, increased and decreased in the livers of the 20‐month‐old *Gdf15* KO mice (Figure 5a and Figure S8e,f). However, no significant difference was found in the naïve and memory CD4^+^ or CD8^+^ T‐cell populations between younger WT and *Gdf15* KO mice (Figure S8g–i). This means that aging can induce mitochondrial stress in cells and that aging stress is required for GDF15 deficiency‐mediated systemic inflammation in mice. Furthermore, Ly6C^+^ inflammatory macrophages and Ly6G^+^ neutrophils were remarkably higher in the livers of 20‐month‐old *Gdf15* KO mice compared with WT controls (Figure S8j,k).

Next, we investigated the functional characteristics of hepatic CD4^+^ and CD8^+^ T cells from 20‐month‐old WT and *Gdf15* KO mice. IFN‐γ production in memory CD4^+^ and CD8^+^ T cells was increased in the liver of 20‐month‐old *Gdf15* KO mice (Figure 5b and Figure S8 l,m). In addition, the population of TNF‐α or IL‐17A‐producing memory CD4^+^ and CD8^+^ T cells was significantly increased in the liver of the old *Gdf15* KO mice (Figure 5b and Figure S8n–p). We also compared T cells in the mesenteric lymph nodes of 20‐month‐old controls and *Gdf15* KO mice. The population of memory/effector CD4^+^ and CD8^+^ T cells was similar in the mesenteric lymph nodes from old *Gdf15* KO and WT mice (Figure S9a–e). Taken together, our data demonstrate that *Gdf15* deficiency induces a larger population of infiltrating pro‐inflammatory immune cells in the livers of older mice.

Diverse innate and adaptive immune cells reside within visceral and subcutaneous adipose tissues in mammals, where they play an essential role in the development of obesity, type 2 diabetes, and metabolic diseases (Lu, Zhao, Meng, & Zhang, 2019). Thus, we investigated the immune cells in the gonadal adipose tissues of 20‐month‐old WT and *Gdf15* KO mice. Adipose CD8^+^ T cells were significantly increased in old *Gdf15* KO mice (Figure S10a,b). The subset analysis of CD4^+^ and CD8^+^ T cells in the gonadal adipose tissue revealed a higher population of memory T cells and a lower population of naïve T cells in old *Gdf15* KO mice (Figure 5c,d and Figure S10c). Moreover, CD11b^+^F4/80^+^ macrophages in gonadal fat were significantly increased in the old *Gdf15* KO mice (Figure S10d). Infiltrating neutrophils and monocytes were remarkably abundant in the gonadal adipose tissues of old *Gdf15* KO mice (Figure 5f and Figure S10e,f). The expression of pro‐inflammatory cytokines and chemokines was also significantly up‐regulated in the gonadal adipose tissues of old *Gdf15* KO mice (Figure 5g). Collectively, *Gdf15* deficiency contributes to the adipose inflammatory response by increasing the population of activated T cells, inflammatory macrophages, and neutrophils in the adipose tissue of aged mice.

### 2.7 GDF15 deficiency deteriorates systemic glucose homeostasis in 20‐month‐old mice

**Figure 6:**
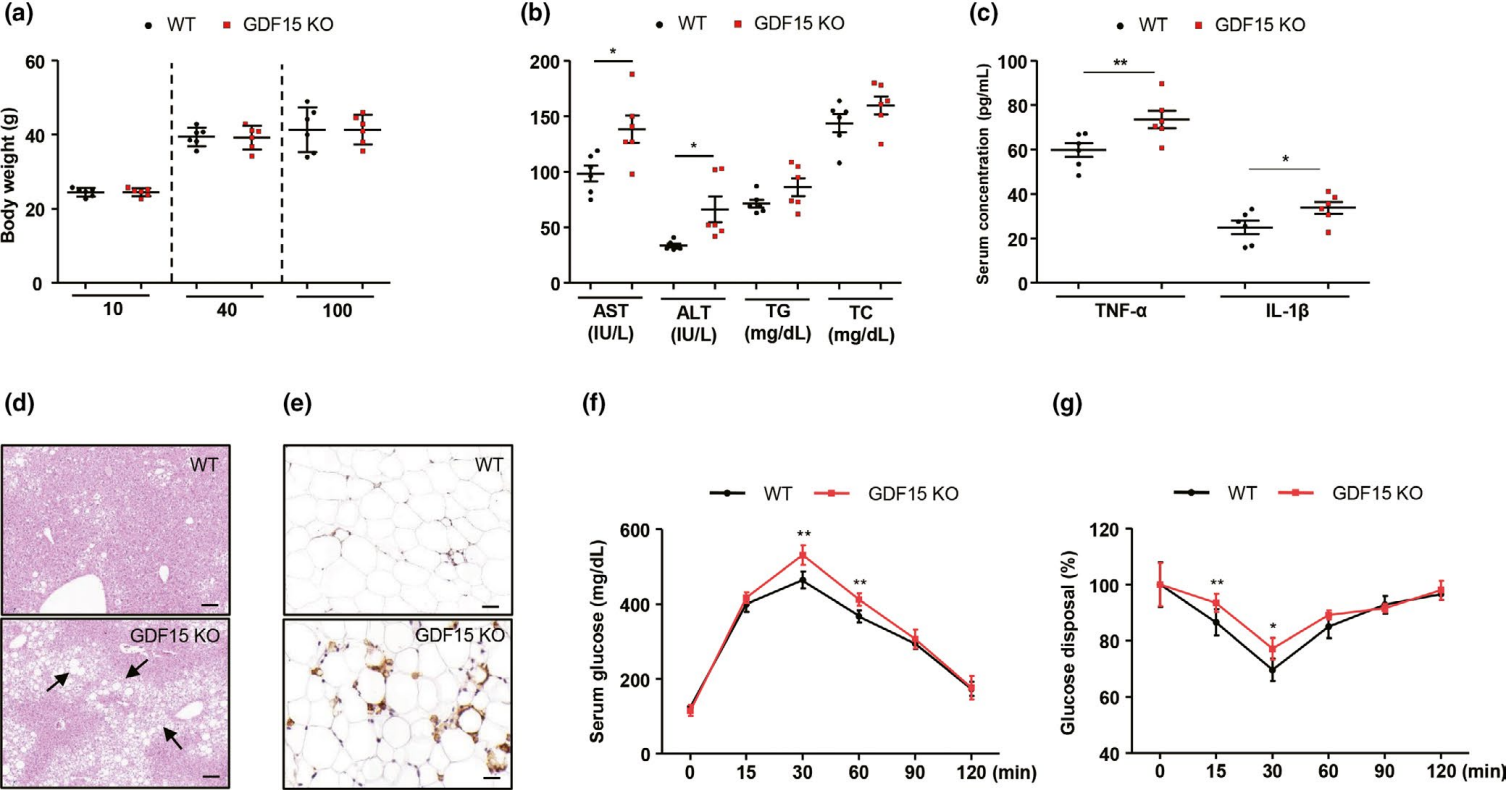
Metabolic phenotyping of WT and *Gdf15* KO 20‐month‐old mice. (a) Body weight of WT and *Gdf15* KO mice at 10‐, 40‐, and 100‐weeks. (b) Serum levels of liver injury markers, triglyceride, and cholesterol profiles of 20‐month‐old WT (n = 6) and *Gdf15* KO (n = 6) mice. (c) Serum levels of pro‐inflammatory cytokines of 20‐month‐old WT (n = 6) and *Gdf15* KO (n = 6) mice. (d) H&E staining for liver tissues of 20‐month‐old WT (n = 6) and *Gdf15* KO (n = 6) mice. Scale bar, 200 μm. Arrows indicate fat accumulation. (e) Fixed adipose tissue from 20‐month‐old WT (n = 6) and *Gdf15* KO (n = 6) mice was stained for F4/80 antibodies. Scale bar, 200 μm. (f) Glucose tolerance tests of 20‐month‐old WT (n = 6) and *Gdf15* KO (n = 6) mice. (g) Blood glucose levels measured over time after intraperitoneal insulin (0.75 U/kg) injection in 20‐month‐old WT (n = 6) and *Gdf15* KO (n = 6) mice. Data (a–c, f, g) are expressed as mean ± SEM. *p < 0.05, **p < 0.01 ((a–c, f,g): two‐tailed t tests)

Next, we studied the impact of *Gdf15* on systemic metabolic homeostasis during the aging process. We found that *Gdf15* depletion did not induce any significant changes in body weight in 10‐ 40‐, or 100‐week‐old mice (Figure 6a). Liver injury markers were increased in the serum of *Gdf15* KO old mice, but serum levels of triglyceride and total cholesterol were similar between WT and *Gdf15* KO old mice (Figure 6b). Serum levels of inflammatory cytokines, including TNF‐α and IL‐1β, were significantly higher in 20‐month‐old *Gdf15* KO mice compared with WT controls (Figure 6c). Moreover, hepatic steatosis (Figure 6d) and liver triglyceride content were elevated in 20‐month‐old *Gdf15* KO mice (Figure S11a). Furthermore, F4/80+ cells were increased in the livers of 20‐month‐old *Gdf15* KO mice (Figure 6e and Figure S11b,c), which also showed higher hepatic expression of pro‐inflammatory cytokines and fibrotic mediators (Figure S11d). Old *Gdf15* KO mice exhibited glucose intolerance compared to age‐matched WT mice during intraperitoneal glucose tolerance tests (Figure 6f). *Gdf15* deficiency also led to significant insulin resistance in 20‐month‐old mice compared with WT mice (Figure 6g). These metabolic phenotypic changes may indirectly affect the increase in memory/effector T cells in the liver and adipose tissues. These data suggest that *Gdf15* protects the mice against aging‐induced glucose intolerance and insulin resistance, as well as tissue inflammation.

### 2.8 Treatment with recombinant GDF15 has no effect on T‐cell activation *in vitro*

**Figure 7:**
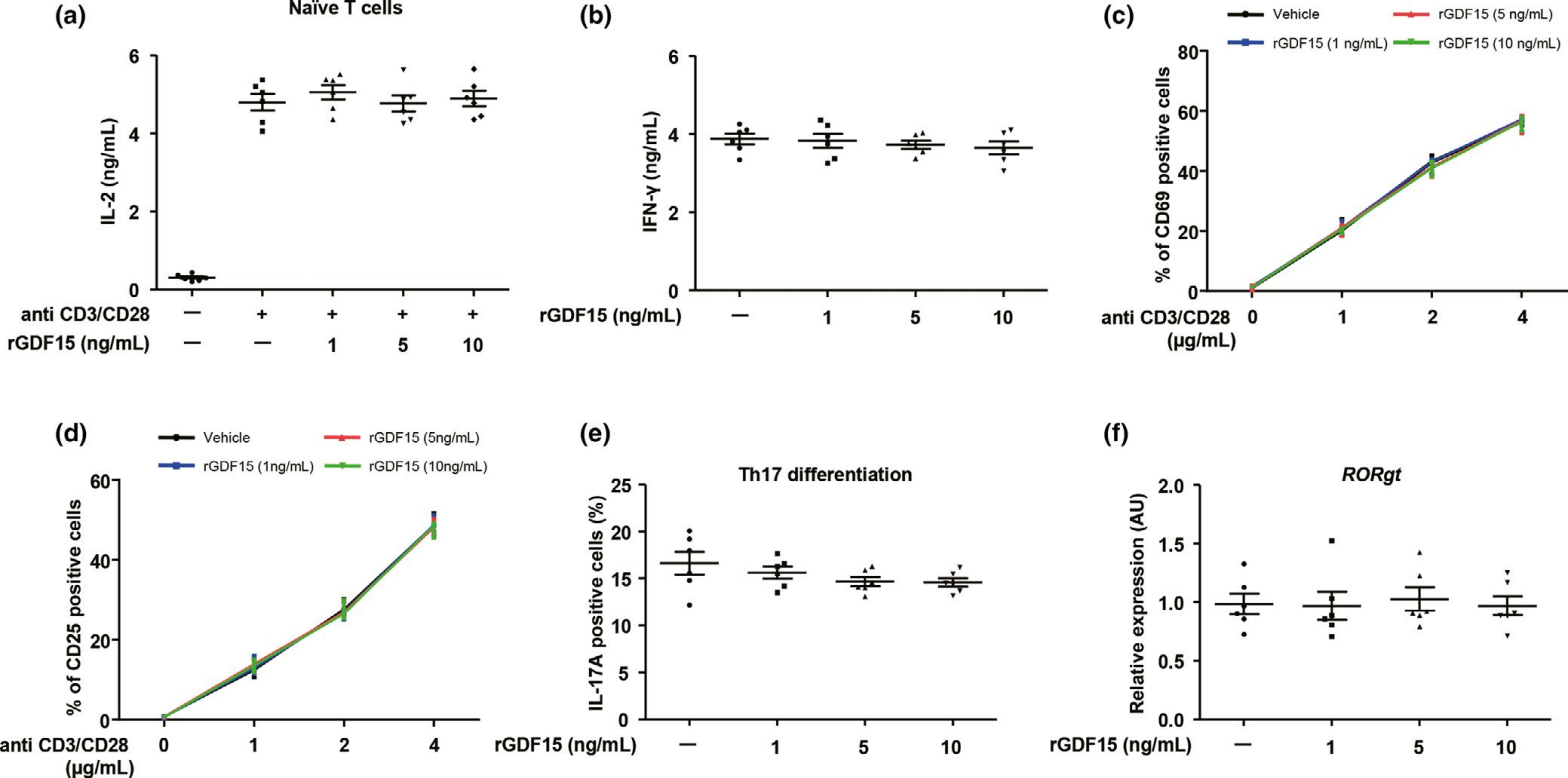
The role of GDF15 in T‐cell activation and Th17 differentiation *in vitro*. (a) IL‐2 production from naïve CD4^+^ T cells stimulated with anti‐CD3 (2 μg/mL)/CD28 (5 μg/mL) in the absence or presence of varying concentrations of recombinant *GDF15* for 72 hr. (b) IFN‐γ production from differentiated CD4^+^ T cells under Th1 culture conditions in the presence or absence of the indicated concentrations of recombinant GDF15. (c,d) Naïve lymph node T cells were stimulated with the indicated concentrations of anti‐CD3/CD28 and several concentrations of recombinant GDF15 for 24 hr and then analyzed for CD69 and CD25 expression by FACS. (e) The population of IL‐17+ cells under Th17 differentiation‐inducing culture conditions at the indicated concentrations of recombinant *GDF15*. (f) The transcription of RORγt in differentiated CD4^+^ T cells under Th17 differentiation‐inducing culture conditions at several concentrations of recombinant GDF15. Data are expressed as mean ± SEM ((a–f): one‐way ANOVA)

To understand how GDF15 contributes to chronic inflammation, we investigated the effect of GDF15 addition on T‐cell activation and Th17 differentiation. Isolated naïve CD4^+^ T cells from human subjects were stimulated with anti‐CD3 and anti‐CD28, which were concomitantly treated with or without several concentrations of recombinant GDF15. The production of IL‐2 and IFN‐γ on CD4^+^ T‐cell activation was not significantly changed by treatment with recombinant GDF15 (Figure 7a,b). Moreover, co‐incubation with recombinant GDF15 did not induce any differences in the number of CD69^+^ or CD25^+^ T cells stimulated with anti‐CD3/CD28 (Figure 7c,d). Furthermore, recombinant GDF15 did not impact on IFN‐γ production upon CD8^+^ T‐cell activation (Figure S12a). IL‐17‐producing effector T helper cells, known as Th17 cells, were implicated in the development of metabolic diseases as well as autoimmune disorders in the elderly (Chehimi, Vidal, & Eljaafari, 2017). Thus, we also assessed the function of GDF15 on Th17 cell differentiation *in vitro*. Th17 cells that were differentiated in the presence of recombinant GDF15 showed similar levels of IL‐17 production compared to the control cells treated with vehicle (Figure 7e). In addition, the expression of RORγt, the key transcriptional regulator for Th17 cell differentiation, was not different between T cells treated with or without recombinant GDF15 (Figure 7f). Furthermore, treatment with recombinant GDF15 did not induce a significant change in oxygen consumption rate and extracellular acidification rate (ECAR) in activated CD4^+^ or CD8^+^ T cells (Figure S12b–e). Collectively, our data show that GDF15 is not required for Th17 cell differentiation from human naïve CD4^+^ T cells and is dispensable for IL‐17 production in Th17 cells.

### 2.9 GDF15 contributes to the regulatory T‐cell‐mediated suppression of conventional T cells

**Figure 8:**
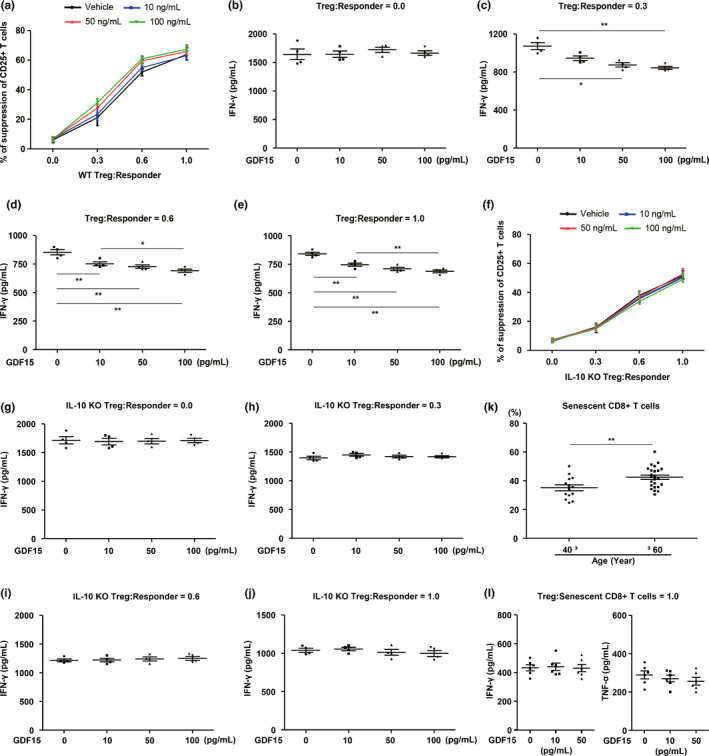
GDF15 enhances Treg‐mediated suppression of T‐cell activation via IL‐10 production in Tregs. (a) Treg‐mediated suppression of the activation (% of suppression of CD25^+^ T cells) of CD4^+^ T cells stimulated with anti‐CD3 (2 μg/mL)/CD28 (5 μg/mL) in the absence or presence of different concentrations of recombinant *GDF15* for 72 hr. (b–e) IFN‐γ levels in supernatants from co‐culturing WT or IL‐10‐deficient Tregs with CD4^+^ T cells stimulated by anti‐CD3 (2 μg/mL)/CD28 (5 μg/mL) at the indicated concentrations of recombinant GDF15 for 72 hr. (f) IL‐10 KO Treg‐mediated suppression of the activation (% of suppression of CD25^+^ T cells) of CD4^+^ T cells in the absence or presence of several concentrations of recombinant GDF15 for 72 hr. (g–j) IL‐10 KO Treg‐mediated suppression of IFN‐γ production of CD4^+^ T cells in the absence or presence of several concentrations of recombinant GDF15 for 72 hr. (k) Population of senescent CD8^+^ T cells in young (**≤**40) and elderly (≥60) human subjects. (l) IFN‐γ or TNF‐α production from senescent CD8^+^ T cells in the absence or presence of several concentrations of recombinant GDF15 for 48 hr. Data are expressed as mean ± SEM. *p < 0.05, **p < 0.01 ((a–h): one‐way ANOVA, (k): two‐tailed t tests, (i–l): one‐way ANOVA)

Although GDF15 is involved in aging‐induced abnormal glucose homeostasis and tissue inflammation in humans and mice, we found that GDF15 does not exhibit a direct role in T‐cell activation and Th17 differentiation. Thus, we investigated whether GDF15 is involved in regulatory T‐cell (Treg)‐mediated inhibition of the effector function of conventional T cells. Effector T cells were co‐cultured with or without Tregs at a ratio of 1:1. Tregs suppressed the proliferation and IFN‐γ production of conventional T cells stimulated with anti‐CD3/CD28 (Figure 8a–e). Then, we co‐cultured Tregs with conventional T cells activated by anti‐CD3/CD28 in the presence of recombinant GDF15. Intriguingly, we found that recombinant GDF15 increased Treg‐mediated suppression of the proliferation and IFN‐γ production of activated T cells *in vitro* (Figure 8a–e). Moreover, we found that recombinant GDF15 increased the expression of *GDNF* family receptor α‐like (GFRAL) in differentiated Tregs (Figure S13). These results indicate that recombinant GDF15 increases Treg‐mediated suppression of conventional T‐cell activation, and thus, likely contributes to the regulation of systemic inflammation.

Moreover, we showed that IL‐10‐deficient Tregs did not manage to increase the suppression of T‐cell activation and failed to reduce IFN‐γ production in activated T cells by treatment with recombinant GDF15 (Figure 8f–j). This suggests that IL‐10 mediates the effect of GDF15 on Treg‐mediated suppression of activated T cells. Additionally, we demonstrated that the population of senescent CD8^+^ T cells within peripheral blood mononuclear cells (PBMCs) was significantly increased in elderly subjects compared with young subjects (Figure 8k). To identify the role of GDF15 on the secretion of inflammatory cytokines from senescent CD8^+^ T cells, we co‐cultured senescent CD8^+^ T cells with Tregs in the presence of recombinant GDF15. Intriguingly, recombinant GDF15 did not induce an increase in Treg‐mediated suppression of IFN‐γ or TNF‐α production in senescent CD8^+^ T cells (Figure 8l). Collectively, these findings suggest that GDF15 increases Treg‐mediated suppression of proliferation and IFN‐γ production in conventional T cells but does not exhibit any role in the regulation of inflammatory cytokines in senescent CD8^+^ T cells.

## 3 DISCUSSION

In this study, we focused on the role of GDF15, a mitochondrial stress‐related secretory factor, in immunometabolic homeostasis during the aging process. We discovered that GDF15‐mediated regulation of tissue‐resident immune cells in the liver and adipose tissues is a key mechanism in preventing the abrupt metabolic deterioration in aging. Thus, we suggest that GDF15 plays an essential role as a new biomarker and candidate drug target for aging and age‐related tissue inflammation.

Although the aging process is complex, mitochondrial stress and dysfunction are universal hallmarks of cellular senescence and aging. Such age‐related mitochondrial damage and dysfunction can result in the leakage of mtDNA in the circulation. Increased concentrations of plasma ccf‐mtDNA have been observed in patients with mitochondrial dysfunction‐related chronic metabolic disorders (Kintscher et al., 2008; Li et al., 2016; Zhang et al., 2010). Here, we found that plasma levels of ccf‐mtDNA were also significantly increased in older subjects and correlated positively with serum levels of GDF15. These findings suggest that aging is a critical factor for the elevation of plasma ccf‐mtDNA levels, which may be derived from mitochondrial stress in humans.

In this study, we found a positive correlation of GDF15 levels with aging in human subjects and in the BXD mouse genetic reference population. In apparent contrast, *Gdf15* KO mice showed an increase in inflammatory and organ damage markers upon aging. This is, however, consistent with a role of the GDF15 mitokine in mitohormesis. During specific periods of life, the secretion of small amounts of mitokines is adaptive as they act to correct and restore some of the underlying mitochondrial abnormalities. We hypothesize that the inherent adaptive nature of mitokine secretion can, however, become maladaptive as uncontrolled mitokine production can contribute to the aging process and disease progression (Khan et al., 2017). Secretion of limited “physiological” levels of mitokines, such as GDF15, may hence correct or cure, through a feedback inhibition, certain of the underpinning cellular abnormalities that lead to their secretion. Higher “supraphysiological” mitokine levels may, however, either contribute to enhance the underlying abnormalities and via a feedforward mechanism enhance disease pathogenesis or reverse the pathology, as observed in the case of FGF21 in the case of mitochondrial myopathy (Khan et al., 2017). In this last scenario, high mitokine levels may also serve as reliable disease biomarkers.

Although the relationship between mitochondrial stress and GDF15 induction is merely correlative and requires further confirmation in follow‐up studies, a large number of previous reports supports the role of GDF15 as a major mitokine regulating metabolic phenotype and inflammatory responses (Choi et al., 2020; Chung, Ryu, et al., 2017; Jung et al., 2018). We also showed previously that GDF15 deficiency exacerbated obesity and glucose intolerance, as well as alcohol‐ and carbon tetrachloride‐induced liver inflammation (Chung, Kim, et al., 2017; Tran, Yang, Gardner, & Xiong, 2018). Adeno‐associated virus expression of human GDF15 (AAV‐hGDF15) also improved body weight and metabolic profiles in diet‐induced obese mice and fat mass remained reduced in 18‐month‐old male B6D2F1 with diet‐induced obese mice, 12 months after injection with AAV‐hGDF15 (Xiong et al., 2017). Recombinant GDF15 furthermore improved the metabolic phenotype in *ob*/*ob* mice (Chung, Ryu, et al., 2017) and inhibited IL‐1β+IFN‐γ‐induced apoptosis of human islets and of a β‐cell line, leading to the prevention of diabetes in NOD mice (Nakayasu et al., 2020). Taken together, GDF15 is a potent hormone responsible for regulating the inflammatory and metabolic phenotypes.

An essential mechanism of immune regulation in tissue inflammation and metabolic diseases involves the action of Tregs (Ilan et al., 2010). In a previous report, Tregs effectively suppressed the pathological and physiological immune responses, leading to immune homeostasis (Miyara & Sakaguchi, 2007). In this study, we found that recombinant GDF15 increased the expression of *Gfral* in differentiated murine Tregs (Figure S13), although further confirmation is needed to define the role of GFRAL in the differentiated Tregs. We also demonstrated that recombinant GDF15 enhances Treg‐mediated suppression of T‐cell activation by increasing IL‐10 production in Tregs. However, senescent CD8^+^ T cells exhibited resistance to Treg‐mediated suppression of IFN‐γ or TNF‐α production in the presence of recombinant GDF15, although senescent T cells exhibited defective T‐cell receptor‐mediated proliferation capacity (Fukushima, Minato, & Hattori, 2018). These findings suggest that aging mediates an increase in the population of senescent T cells and may contribute to the development of systemic inflammation by avoiding Treg‐mediated suppression. Thus, even if GDF15 increases with age, it may be difficult to regulate tissue inflammation and to prevent metabolic disorders in humans and mice.

Aging is also associated with chronic inflammation in several tissues (Sanada et al., 2018). Lipid accumulation and infiltration of activated T cells, monocytes/macrophages, or neutrophils into metabolic organs increase tissue inflammation and dysregulation of metabolic homeostasis (Byun & Yi, 2017; Kintscher et al., 2008; Mirmiran, Bahadoran, & Azizi, 2014). In contrast, the depletion of these inflammatory immune cells ameliorates systemic inflammation and insulin resistance (Jung et al., 2018; Yang et al., 2010). Moreover, T‐cell aging is involved in glucose intolerance and insulin resistance in humans and mice (Yi et al., 2019). In this study, we demonstrated that *Gdf15* deficiency enhanced the infiltration of effector T cells and inflammatory macrophages into the liver and adipose tissues, accentuating liver injury, and insulin resistance in aged mice. Although we have not demonstrated an effect of recombinant GDF15 on inflammatory or metabolic phenotypes in old mice, previous studies (Choi et al., 2020; Chung, Kim, et al., 2017; Chung, Ryu, et al., 2017; Tran et al., 2018) support the idea that GDF15 can regulate metabolic homeostasis by modulating the tissue inflammatory response in various situations. However, further study is required to establish a functional role for GDF15 in aging‐mediated inflammation and metabolic diseases in humans.

Although recent studies emphasized the importance of the *GDNF* family receptor α‐like (GFRAL)/GDF15 signaling in hindbrain neurons (Emmerson et al., 2017; Mullican et al., 2017; Yang et al., 2017), previous investigations also showed peripheral effects of GDF15 (Chung, Ryu, et al., 2017; Kim et al., 2018; Lee et al., 2017). Moreover, treatment with recombinant GDF15 ameliorated non‐alcoholic or alcoholic fatty liver in mice, without affecting food consumption (Chung, Ryu, et al., 2017; Kim et al., 2018). In the current study, we utilized the BXD mouse reference population and GTEx human transcriptomic data to unravel a network of GDF15‐related genes and phenotypes in different human (GTEx) and mouse (BXD) tissues under basal physiological conditions. *GDF15* expression increased during normal aging in the mouse BXD population and in two independent human cohorts. Moreover, we demonstrated that aged mice with a higher hepatic *Gdf15* transcript levels had decreased health and lifespan, despite the absence of changes in food intake. Combined, these data may suggest a role of GDF15 in the regulation of survival and aging‐related metabolic diseases through a peripheral mechanism independent of GFRAL. However, further studies are needed to establish peripheral effects of GDF15‐GFRAL axis using tissue‐specific GFRAL knockout animal models.

In conclusion, GDF15 is induced by mitochondrial dysfunction and systemic inflammation in humans and mice during the aging process. Our study also reveals critical insights into the regulation of tissue inflammation by GDF15 during the aging process. Although previous studies showed that recombinant GDF15 improves the metabolic phenotype and tissue inflammation in mouse models, additional mechanistic studies will be needed to confirm GDF15 supplementation is a potential therapy against age‐related chronic diseases.

## 4 EXPERIMENTAL PROCEDURES

### 4.1 Human subjects

Peripheral blood samples (10 ml each) were obtained from 70 study participants in the Department of Endocrinology and Metabolism outpatient clinic at Chungnam National University Hospital (CNUH). Human subjects with any of the following conditions were excluded from the study: previous coronary heart disease, uncontrolled hypertension, chronic obstructive pulmonary disease, acute or chronic kidney disease (estimated glomerular filtration rate < 30 ml/min/1.73 m^2^), anemia (hemoglobin < 12 g/dl), history of any malignant or inflammatory disease, current liver disease, or high plasma aspartate transaminase or alanine transaminase (>80 IU/L). Liver tissues were collected from 30 subjects who underwent lobectomy or segmentectomy at CNUH. The non‐tumor areas in the liver were isolated and used for real‐time PCR analysis. Baseline clinical characteristics of the study subjects are described in Table S1. Prior to their inclusion in this study, written informed consent was obtained from all participants. This study was also approved by the Institutional Review Board of CNUH (CNUH 2015‐09‐042; CNUH 2019‐06‐063). All experiments were performed in accordance with the standards of the Declaration of Helsinki and related guidelines.

### 4.2 Mice

Male C57BL/6 WT mice were purchased from Jackson Laboratory (Bar Harbor, ME, USA), and *Gdf15* KO mice were provided by S. Lee, Johns Hopkins University School of Medicine (Baltimore, MD). IL‐10‐deficient mice were purchased from the Jackson Laboratory (Bar Harbor, ME) and were backcrossed with the B6 strain for more than ten generations. To generate skeletal muscle‐specific and adipocyte‐specific *Crif1* knockout mice, floxed Crif1 mice were crossed with *Adipoq*‐*Cre* mice (a kind gift from E. Rosen, Beth Israel Deaconess Medical Center, Boston, MA, USA) or *Mlc1f*‐*Cre* mice on a C57BL/6 background. All mice were housed in a controlled environment (12‐h light/12‐h dark cycle; humidity: 50%–60%; ambient temperature: 22 ± 2°C) and fed a normal chow diet in a specific pathogen‐free animal facility at the CNUH Preclinical Research Center. To avoid any possible effects of estrogen on GDF15 production, all *in vivo* experiments were conducted on male mice. WT and *Gdf15* KO old (20‐month‐old) mice were used to investigate the effect of GDF15 on aging‐induced systemic inflammation. All animals received humane care according to institutional guidelines, and all experiments were approved by the Institutional Review Board of CNUH. All represented BXD phenotypes in this study were reanalyzed using publicly available datasets at the GeneNetwork website (http://www.genenetwork.org and http://gn2.genenetwork.org; GeneNetwork accession numbers: GN843 and GN859) (Andreux et al., 2012).

### 4.3 FACS analysis

Hepatic and adipose immune cells were pre‐incubated with anti‐mouse CD16/32 Fc blocker (BD Pharmingen, USA), followed by staining with the Live/Dead marker anti‐FVD‐APC‐Cy7 (all supplied by eBioscience, San Diego, CA, USA). The fluorochrome‐conjugated antibodies used in this study were anti‐CD45, anti‐CD3, anti‐NK1.1, anti‐CD4, anti‐CD8, anti‐CD44, anti‐CD62L, anti‐CD11b, anti‐F4/80, anti‐Ly6C, anti‐Ly6C, and anti‐Siglec‐F (all supplied by eBioscience, San Diego, CA, USA). Liver mononuclear cells were stimulated with phorbol‐myristate acetate/ionomycin/brefeldin A/monensin for 5 hr *in vitro*. The cells were fixed and permeabilized using a Fixation/Permeabilization Buffer kit (eBioscience, San Diego, CA, USA). The permeabilized cells were washed with FACS buffer and resuspended in 1% formaldehyde and stained for intracellular cytokines with anti‐IFN‐γ‐PE‐Cy7, anti‐TNF‐α‐APC, and anti‐IL‐17A‐APC fluorochrome‐conjugated antibodies. Stained cells were analyzed using a BD LSRFortessa flow cytometer (BD Biosciences, San Jose, CA, USA), and data were analyzed using FlowJo software (FlowJo, LLC, Ashland, OR, USA).

### 4.4 Bioinformatics analysis of the Innocenti et al. dataset

We obtained the liver gene expression microarray dataset of a previous report (Innocenti et al., 2011) from the GEO database at https://www.ncbi.nlm.nih.gov/geo/query/acc.cgi?acc=GSE25935. The dataset contains 464 unique Agilent gene expression microarrays *m* corresponding to healthy human liver biopsies from 206 patients *p*. Available covariates include patients’ age, gender, and ancestry, defined as the first principal component in the analysis of Innocenti et al., which separated African from non‐African individuals. The 16 samples obtained from patients 325, 333, 716, and 720 were removed from the data because of inconsistencies in their age and gender annotations.

The intensities are already preprocessed: Intensities were log_2_‐transformed, background subtracted with the minimum method (Ritchie et al., 2007), and quantile normalized (Bolstad, Irizarry, Astrand, & Speed, 2003). For each gene *i*, we assumed the following linear regression model:

$$y_{\mathrm{im}}=\beta_{i}^{0}+x_{a,pm}\beta_{i}^{\text{age}}+x_{a,pm}^{2}\beta_{i}^{{\text{age}}^{2}}+x_{b,pm}\beta_{i}^{\text{ancestry}}+\beta_{\mathrm{ig}}^{\text{gender}}+u_{\mathrm{ip}}^{\text{patient}}+\varepsilon_{\mathrm{im}}.$$

where *y_im_* is the preprocessed log_2_ intensity of gene *i* in sample *m*, $\beta_{i}^{0}$ is the intercept, $\beta_{i}^{\text{age}}$ and $\beta_{i}^{\text{ancestry}}$ are the effects of age and ancestry, respectively, and $\beta_{i}^{{\text{age}}^{2}}$ is a quadratic effect for age. This quadratic effect allows us to capture non‐linear trends in the data. *x_a_*_,_ *_pm_* and *x_b_*_,_ *_pm_* are vectors of length 448 wherein each element corresponds to, respectively, the age and ancestry of patient *p* in sample *m*.

$\beta_{\mathrm{ig}}^{\text{gender}}$ is a dummy variable which is equal to 0 if the sample is from a male patient and equal to 1 if the sample is from a female patient. $u_{\mathrm{ip}}^{\text{patient}}$ is a random patient effect that accounts for the fact that samples originating from the same patient are correlated. $u_{\mathrm{ip}}^{\text{patient}}$ is assumed to be normally distributed ($u_{\mathrm{ip}}^{\text{patient}}∼N\left(0,\sigma_{u_{i}}^{2}\right)$). $\varepsilon_{\mathrm{im}}∼N\left(0,\sigma_{i}^{2}\right)$ is a random error term. Statistical significance was assessed with the limma R package (Smyth, 2004), whereby inter‐technical replicate variation was estimated by fitting gene‐wise linear mixed models with the duplicateCorrelation function, as previously described (Smyth, Michaud, & Scott, 2005). We used limma's empirical Bayes‐moderated F statistic to test the combined significance of the linear and quadratic age effects and corrected for multiple testing with the Benjamini–Hochberg FDR procedure.

### 4.5 Bioinformatics analysis of the Genotype‐Tissue Expression (GTEx) dataset

The Auwerx lab has access to the Genotype‐Tissue Expression (GTEx) Project version 8 through dbGaP accession number phs000424.v8.p2 (Project #10143 AgingX). To study the relation between age and *GDF15* expression, we assessed the raw RNA‐Seq gene count data from the livers of 226 deceased individuals. For the statistical analysis, we removed three persons for which no information on smoking and drinking status was known and one person for which no information on cocaine usage was known. Effective library sizes were calculated based on trimmed mean of M value (TMM) normalization factors (Robinson & Oshlack, 2010) as implemented in edgeR (Robinson, McCarthy, & Smyth, 2010). To account for over‐excess zero counts in low‐expressed genes, we fitted with a zero‐inflated negative binomial model as previously described (Van den Berge et al., 2018erge et al., 2018) and implemented in the zingeR R package. Sample‐level covariates included a linear and quadratic effect for age, a linear and quadratic effect for the sample's ischemic time, and dummy variables to account for the effects of gender, sample collection site, smoking status, drinking status, and cocaine usage in the past five years. The combined significance of the linear and quadratic age terms was inferred with an F test with adjusted denominator degrees of freedom. Multiple testing was controlled by DESeq2’s independent filtering procedure (Love, Huber, & Anders, 2014) followed by Benjamini–Hochberg FDR correction.

To further explore the role of *GDF15* expression in different tissues (liver, subcutaneous adipose tissue, visceral adipose tissue, and muscle), the expression profiles of GTEx version 8 were obtained from the GTEx Portal (https://gtexportal.org/). We identified differentially expressed genes by establishing two groups based on the *GDF15* expression level of each tissue with the R package DESeq2 (Love et al., 2014). The gene‐set collection of KEGG was obtained from Enrichr (https://amp.pharm.mssm.edu/Enrichr/), and the gene‐set enrichment analysis was conducted with the R package PIANO, by using the DEA results taken from DESeq2. Differentially expressed genes (DEG) with *p*‐value <.05 and KEGG pathways with Benjamini–Hochberg‐corrected FDR values <0.1 were considered statistically significant. The correlation analysis of *GDF15* and individual genes was done by Gene Expression Profiling Interactive Analysis (GEPIA; http://gepia2.cancer‐pku.cn/). The full list of significantly enriched pathways in the liver, subcutaneous adipose tissue, and skeletal muscle i included in Tables [Link], [Link], [Link].

### 4.6 Mendelian randomization

We used the publicly available GTEx cis‐eQTL data in whole blood as exposure effect estimates. After clumping based on linkage disequilibrium with the ld_clump function from the ieugwasr v 0.1.4 R package (https://mrcieu.github.io/ieugwasr/), one single nucleotide polymorphism (SNP), rs7226, was retained. rs7226 has been linked to GDF15 concentration in blood before (Jiang et al., 2018) and is located ~12 kb upstream of *GDF15* inside the fifth and last exon of *PGPEP1*, a gene that is known to be co‐expressed with *GDF15* (Ho et al., 2012). The estimated effects of rs7226 on 33 obesity‐related outcomes and 27 outcomes related to blood leukocyte concentrations (Table S2) were obtained from the IEU GWAS database (https://gwas.mrcieu.ac.uk/datasets/) (Hemani et al., 2018) through the available_outcomes function from the TwoSampleMR v 0.5.3 R package (https://github.com/MRCIEU/TwoSampleMR). We used the same package to calculate Wald ratios of the outcome effect estimates to the exposure effect estimate. The p‐values were converted to q‐values with the Benjamini–Hochberg multiple testing procedure.

### 4.7 Tregs‐mediated suppression of conventional or senescent T cells

Isolated CD4^+^CD25^−^ T cells were labeled with carboxyfluorescein succinimidyl ester (CFSE) (Invitrogen, Eugene, OR, USA) just before co‐culturing with Tregs following the manufacture's guidelines. CFSE‐labeled CD4^+^CD25^−^ T cells were activated with anti‐CD3 (2 μg/mL)/CD28 (5 μg/mL) in the presence of different amounts of Tregs from C57BL/6 mice. The suppressive capacity of Tregs was measured by the addition of Tregs at Treg:Teff ratios of 0.25:1, 0.5:1, and 1:1 in 96‐well round‐bottom plates in the absence or presence of the indicated concentrations of recombinant GDF15 for 72 hr. CD8^+^CD28^−^ T cells were sorted by FACS Aria II (BD Bioscience, San Jose, CA, USA) using surface immunofluorescence‐conjugated antibodies and were labeled with CFSE. To assess the suppressive capacity of Tregs, the CD8^+^CD28^−^ T cells were also activated with anti‐CD3 (2 μg/ml)/CD28 (5 μg/ml) in the presence of different amounts of Tregs under the indicated concentrations of recombinant GDF15. Then, IFN‐γ levels from the culture supernatants were measured, and the relative suppression of proliferation was determined by assessing the inhibition of CFSE dilution.

### 4.8 Statistical analysis

All continuous variables are reported as the mean ± SE of the mean, except if explicitly stated otherwise. Statistical analyses were performed using GraphPad PRISM software (GraphPad, San Diego, CA, USA). All data from the mouse studies were analyzed by two‐way repeated‐measures ANOVA followed by Bonferroni's multiple comparison, a one‐way ANOVA followed by Tukey's *post hoc* test, or a two‐tailed Student's t test. Statistical correlations were evaluated using Pearson's correlation coefficient. *p* values < 0.05 were considered statistically significant.

## CONFLICT OF INTEREST

No potential conflicts of interest are reported.

## AUTHOR CONTRIBUTIONS

J.S.M, D.R., and H.Y. designed research and analyzed data. J.S.M, J.W.T., H.T.N., and S.G.K carried out all experiments. L.J.E.G., B.E.K., and J.A. helped with the omics studies. S.K., M.S., and J.A. provided critical scientific insights. J.T.K., L.J.E.G., J.A., D.R., and H.Y. wrote the manuscript. J.S.B, Y.L., and J.J. provided helpful discussion.

## Supporting information

Figure S1‐S13

Table S1

Table S2

Table S3

Table S4

Table S5

Table S6

Supinfo

## Data Availability

The data that support the findings of this study are available from the corresponding author upon reasonable request.
