# Supplementary figures and images for: Growth differentiation factor 15 protects against the aging‐mediated systemic inflammatory response in humans and mice
Source: Aging Cell. 2020 Jul 21;19(8):e13195. doi: 10.1111/acel.13195 (PMC7431835; doi:10.1111/acel.13195)

(a)

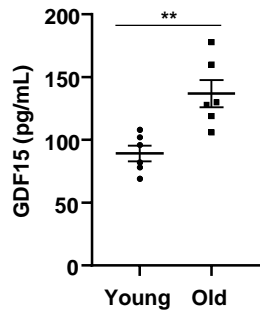

(b)

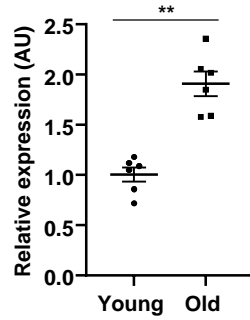

(c)

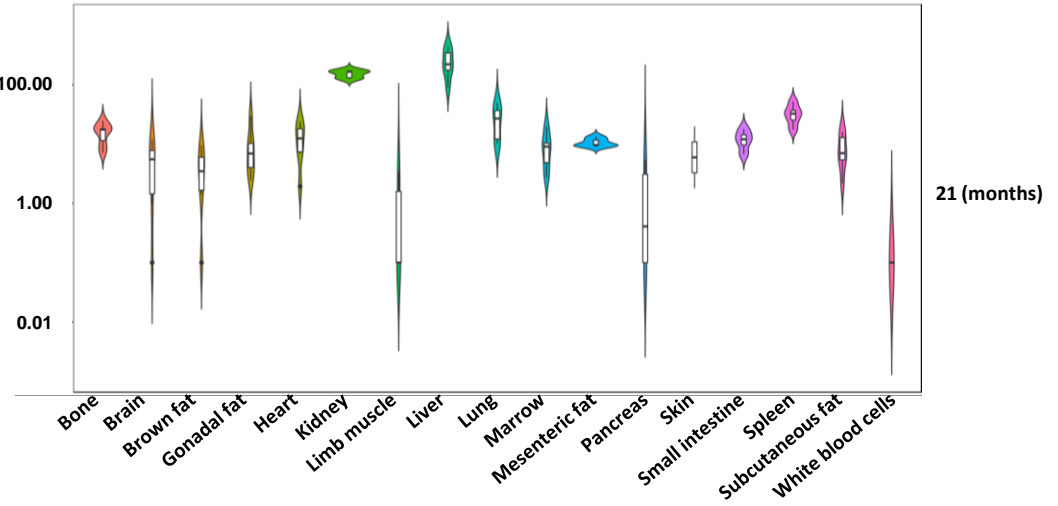

(d)

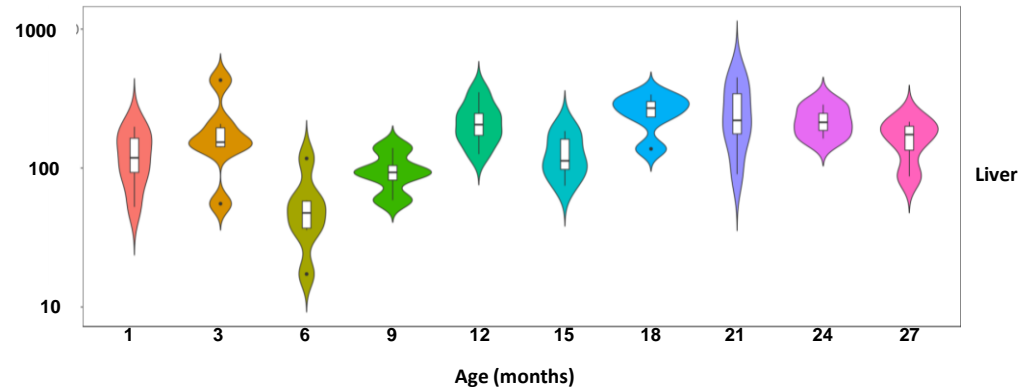

(e)

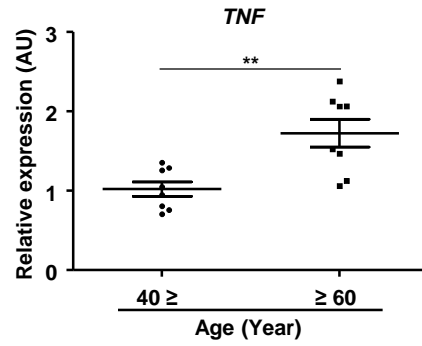

(f)

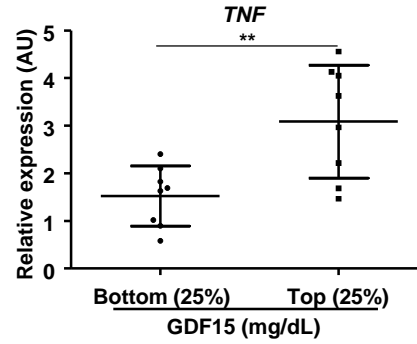

(g)

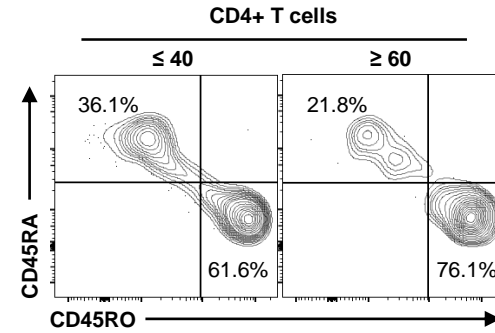

(h)

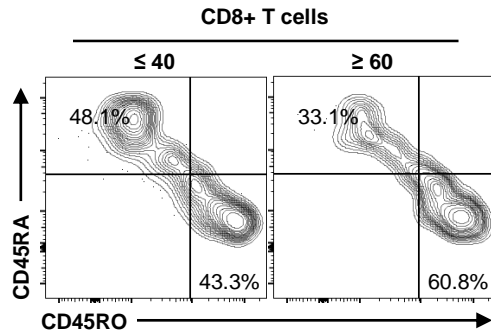

(i)

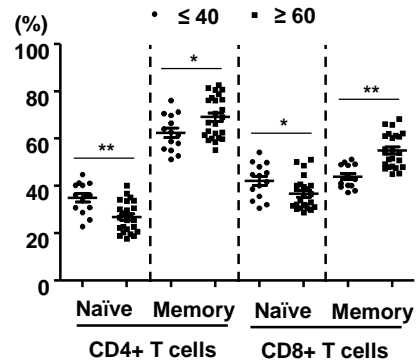

(j)

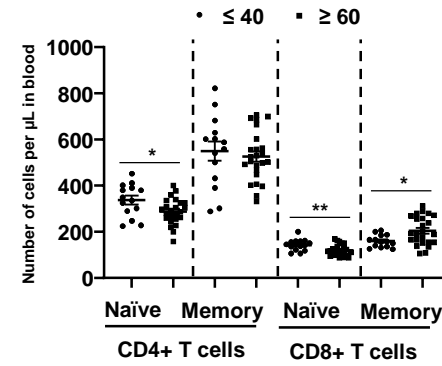

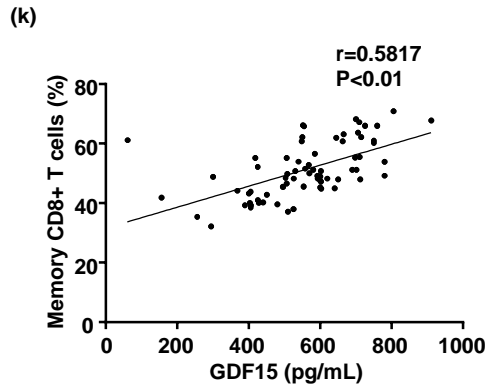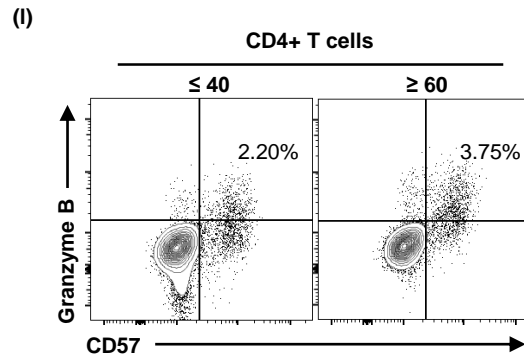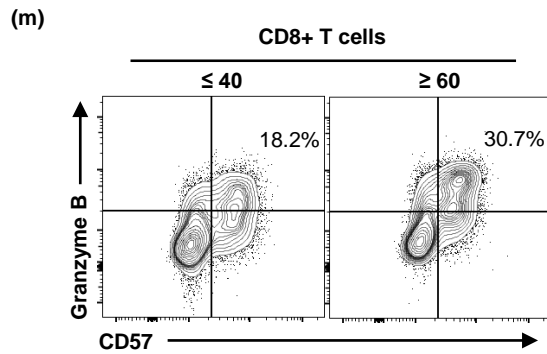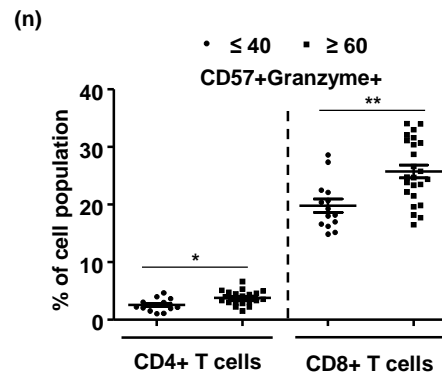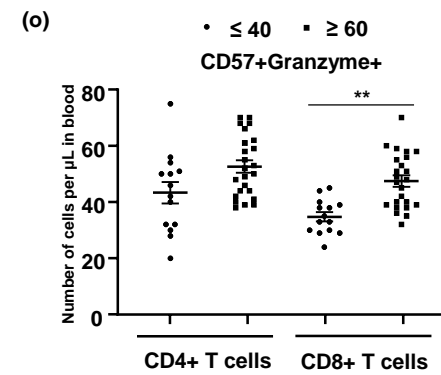

(a)

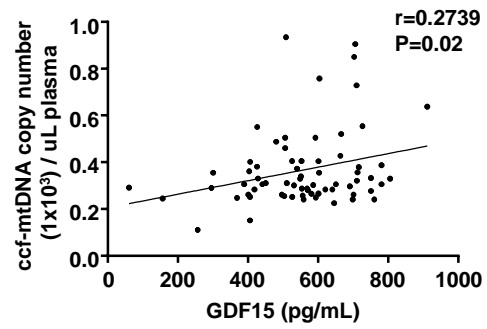

(b)

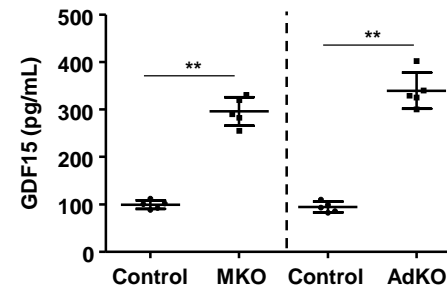

(a)

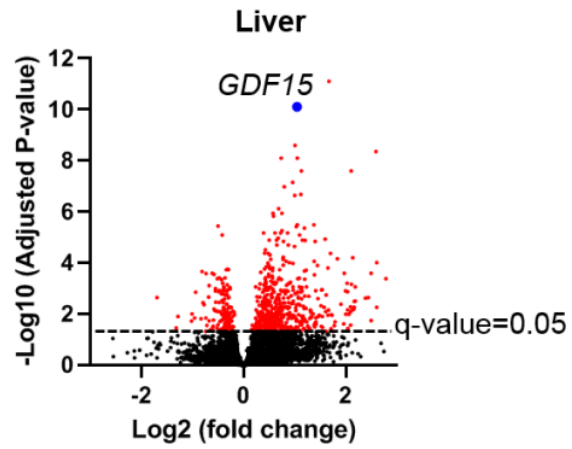

(b)

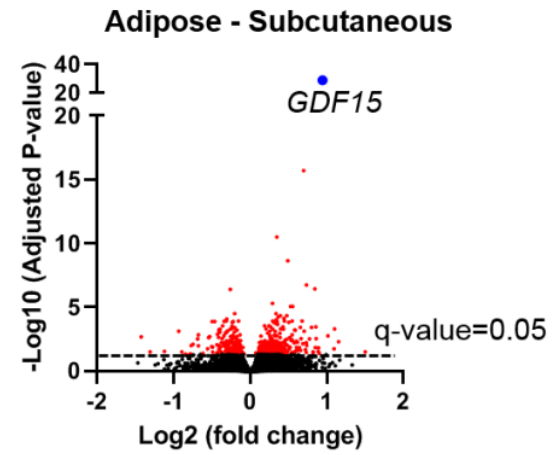



(a)

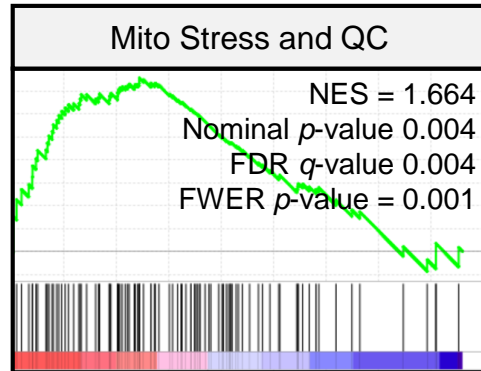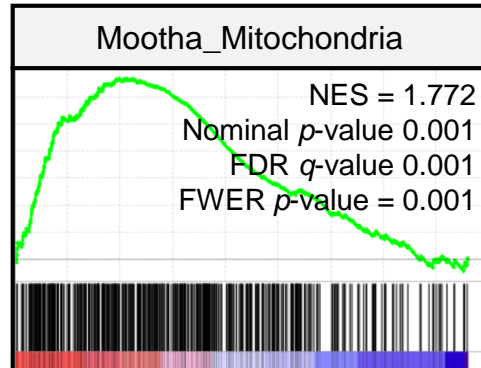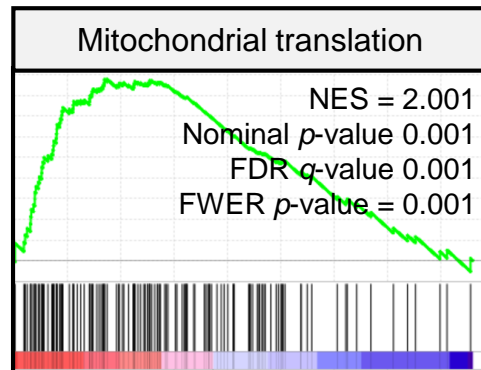

Gdf15 hi(25%)      Gdf15 low(25%)

(b)

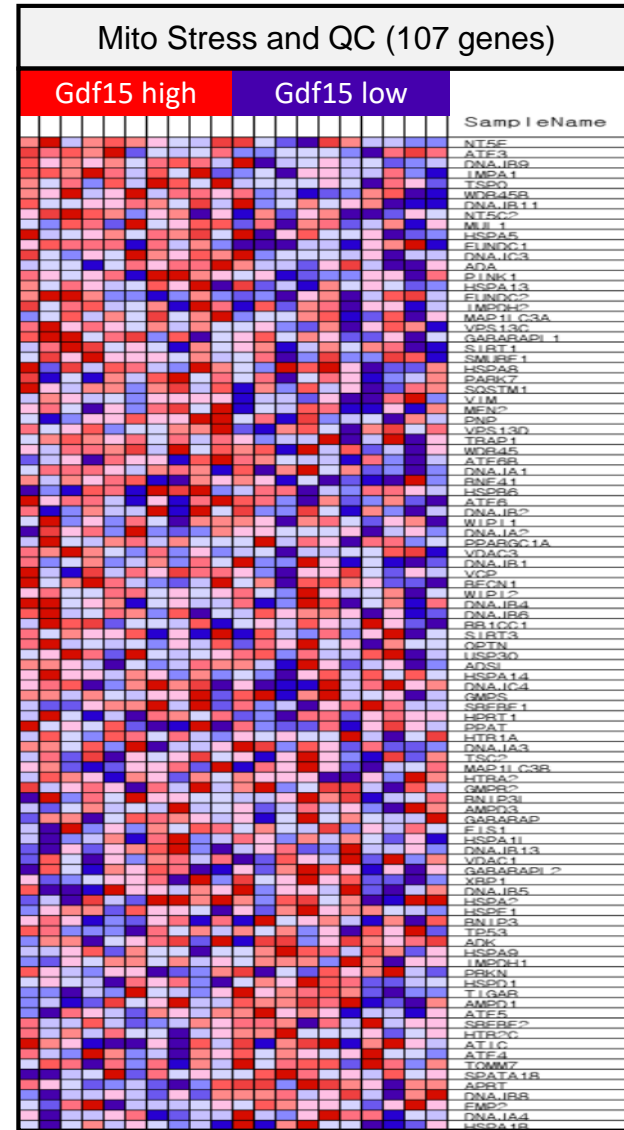

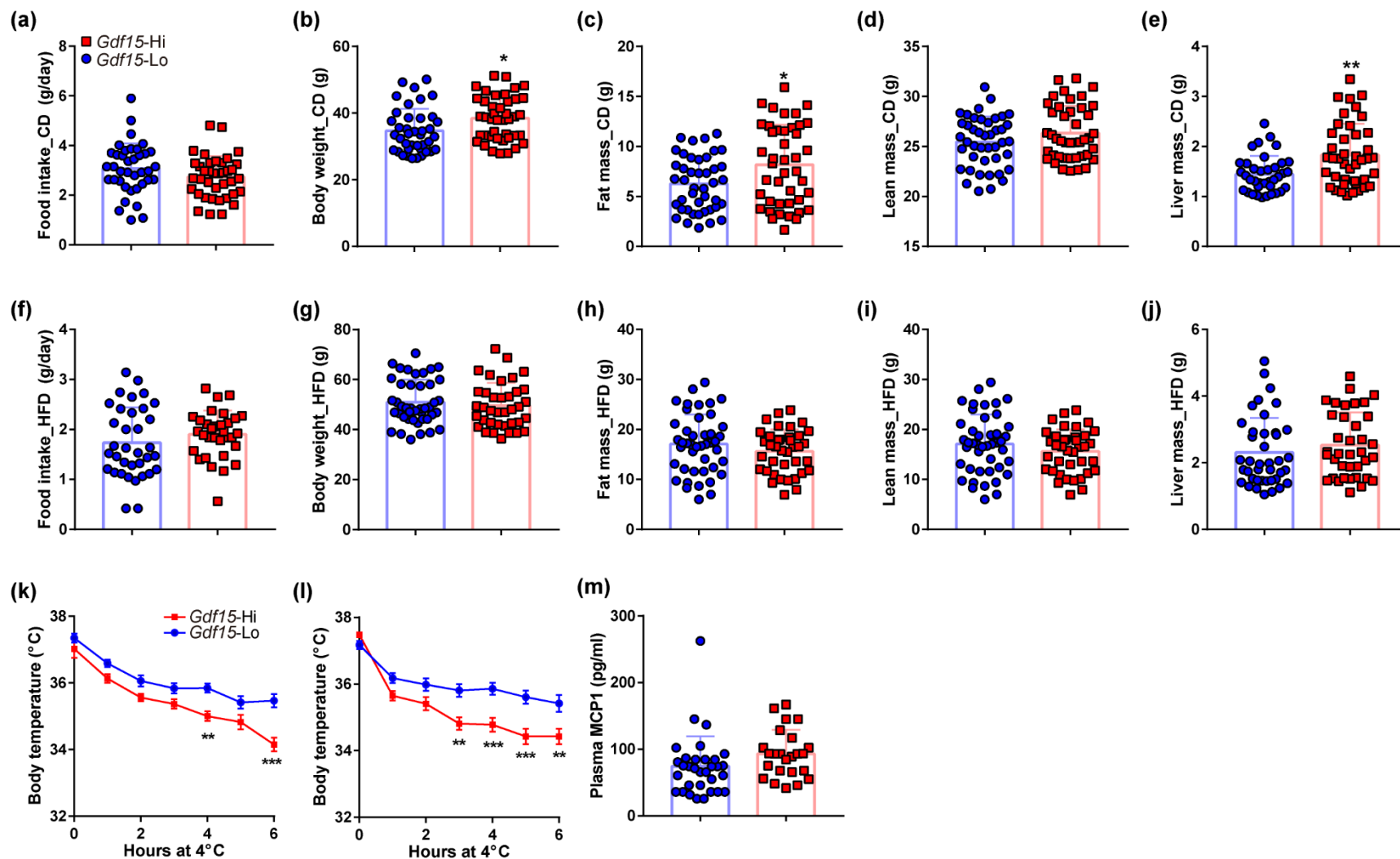

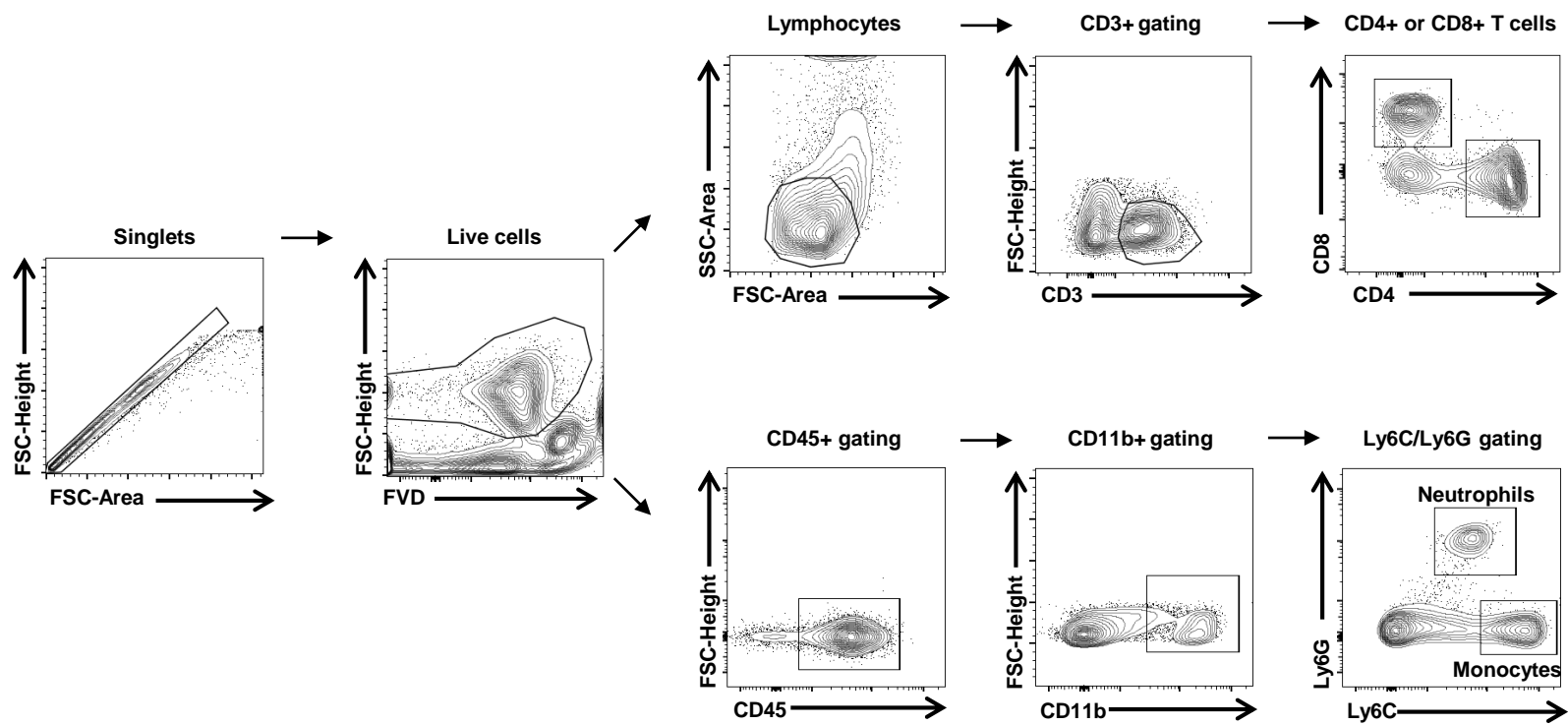

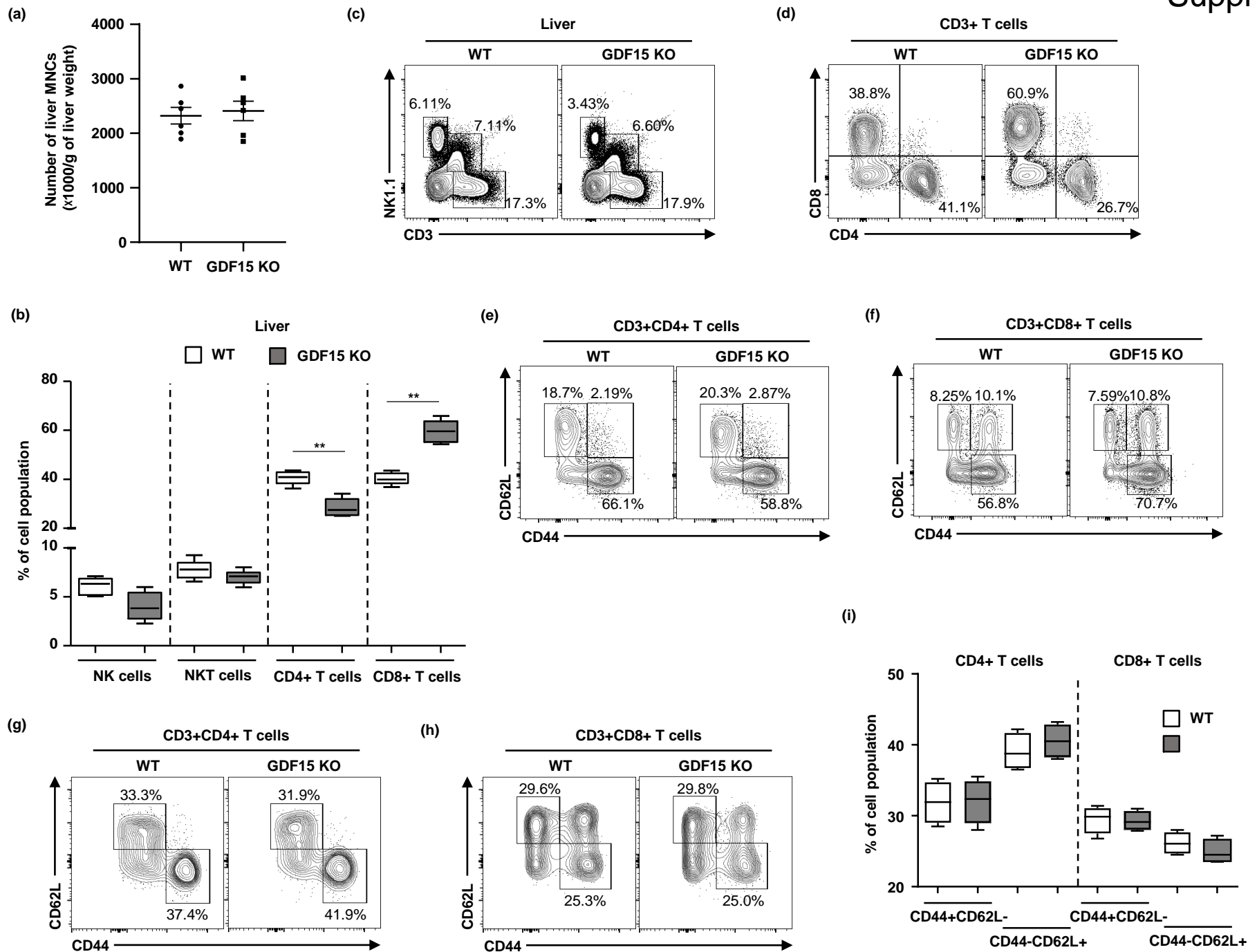

(j)

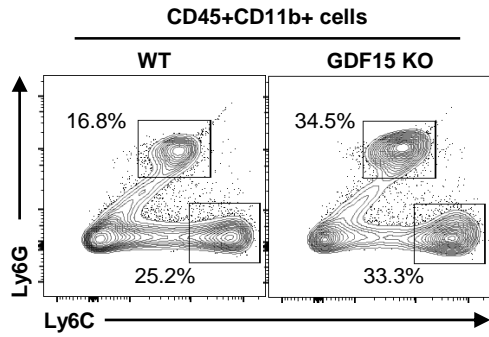

(k)

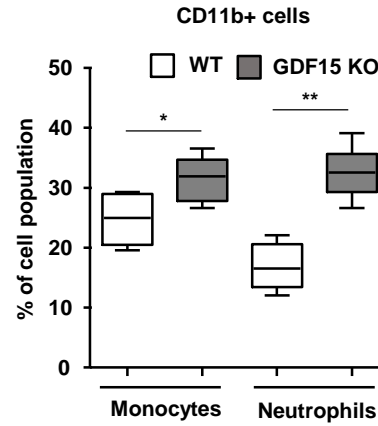

(l)

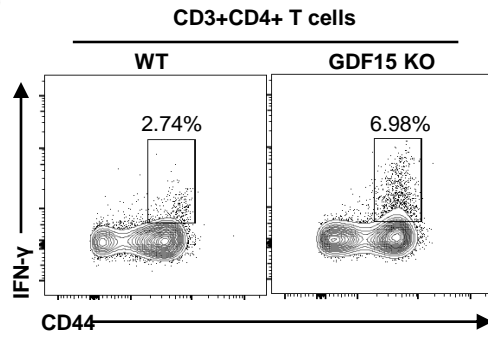

(n)

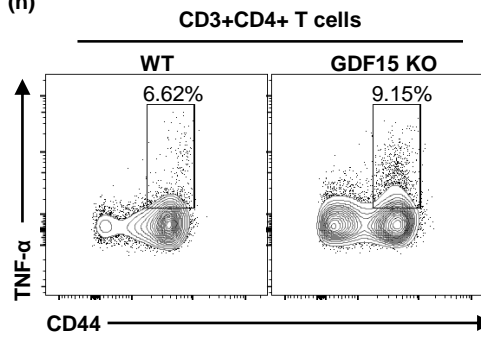

(m)

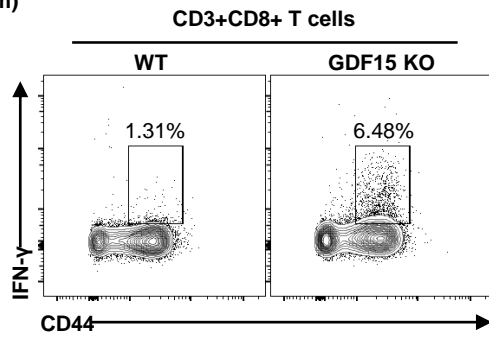

(o)

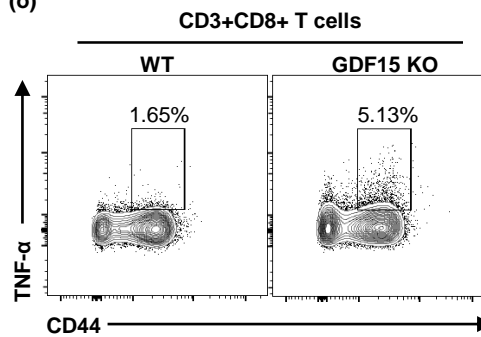

(p)

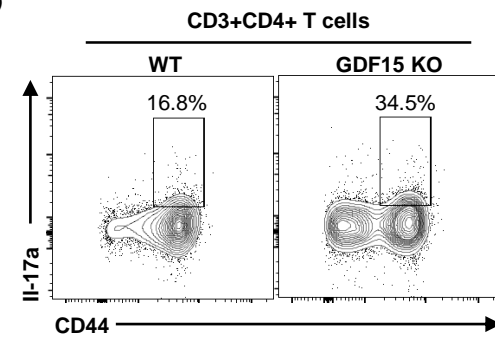

(a)

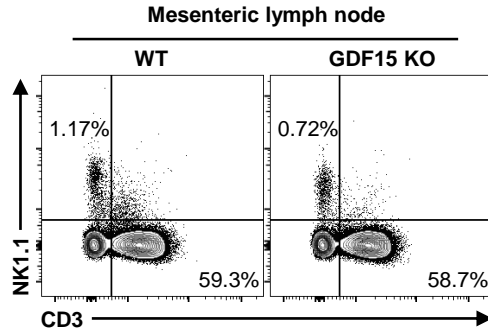

(b)

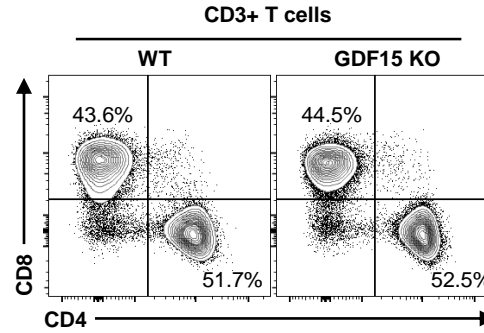

(c)

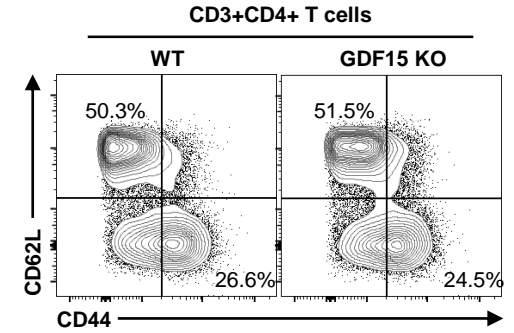

(d)

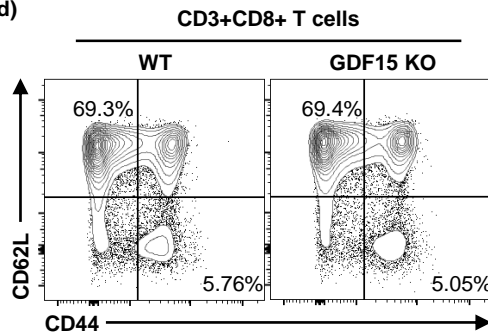

(e)

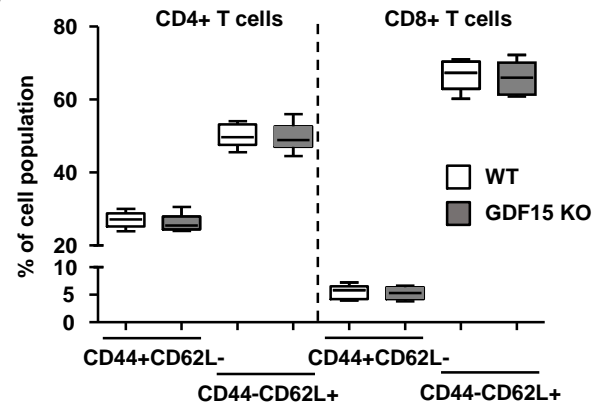

(a)

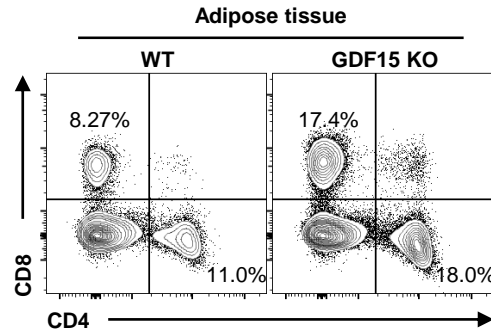

(b)

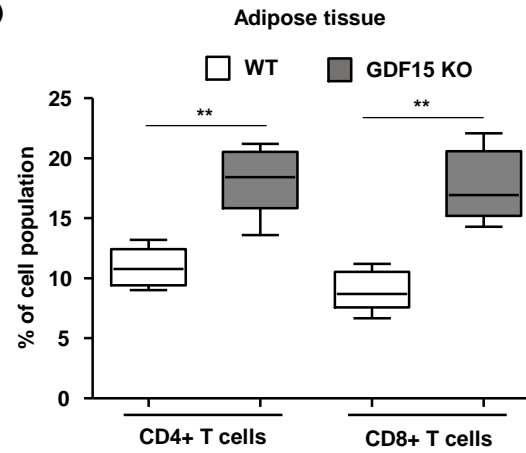

(c)

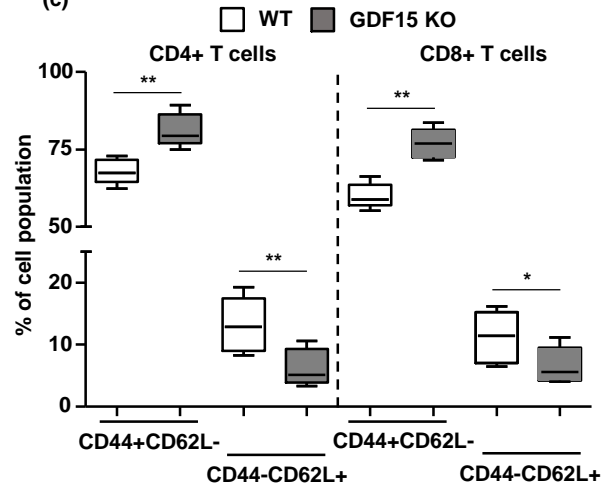

(d)

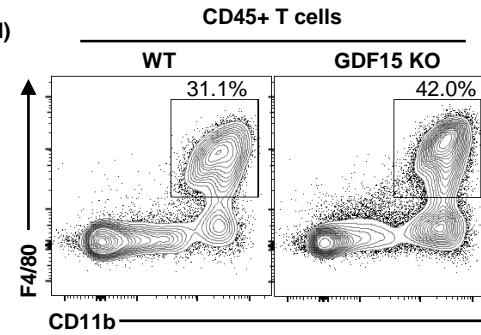

(e)

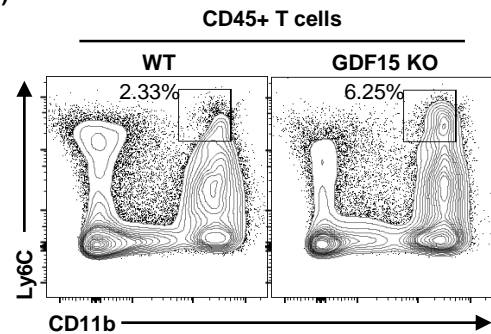

(f)

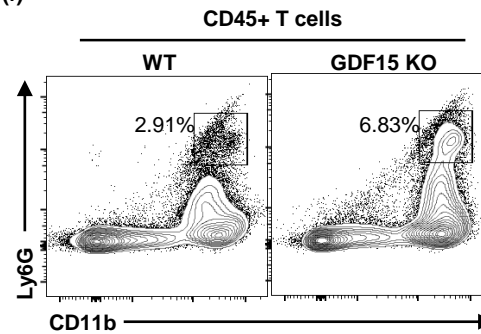

(a)

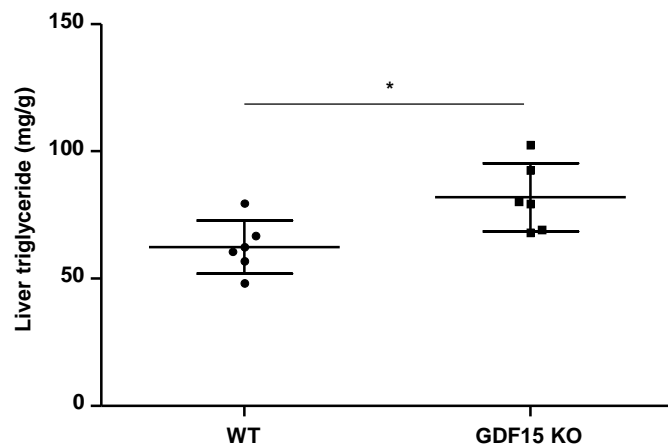

(c)

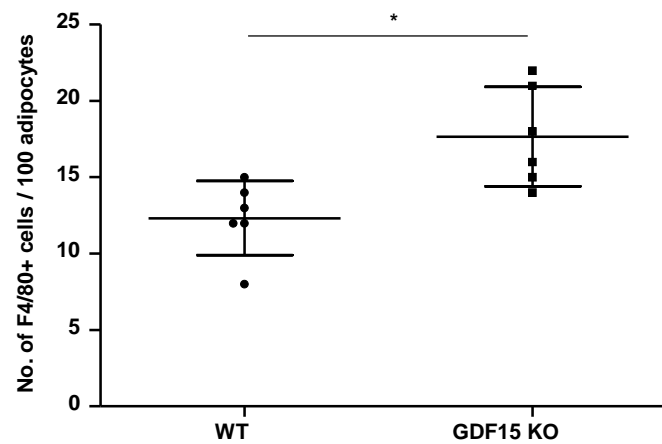

(b)

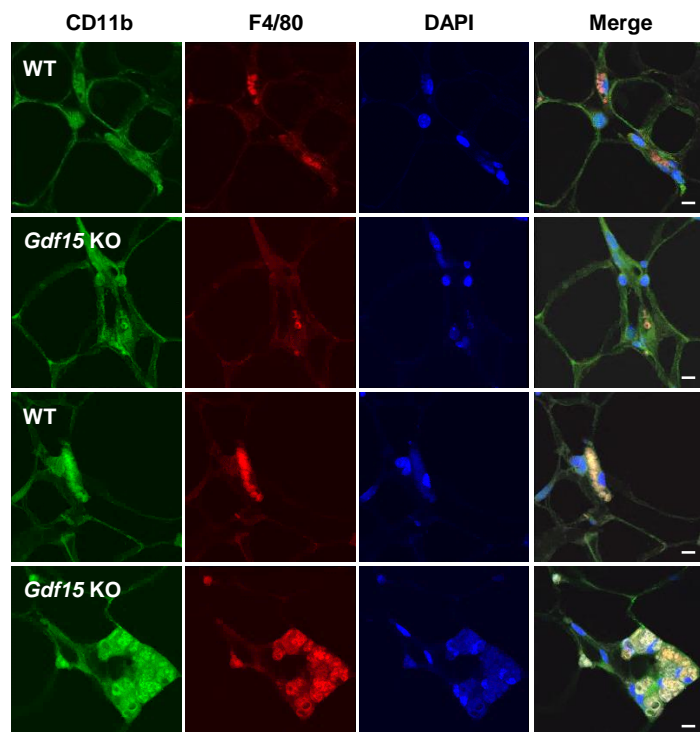

(d)

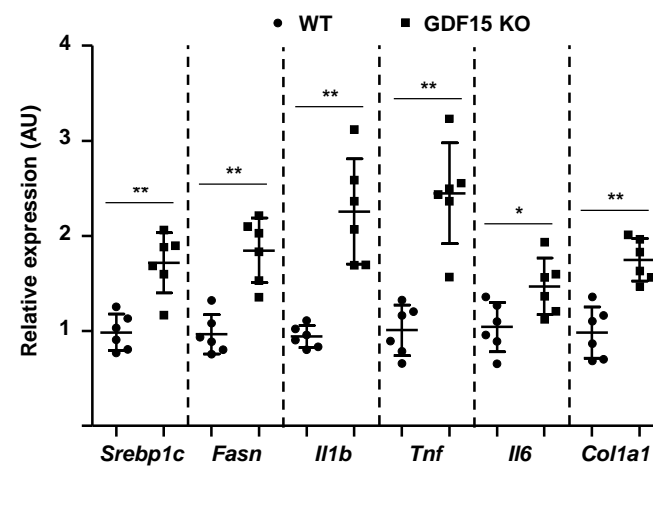

● Vehicle ○ rGDF15 (10ng/mL)

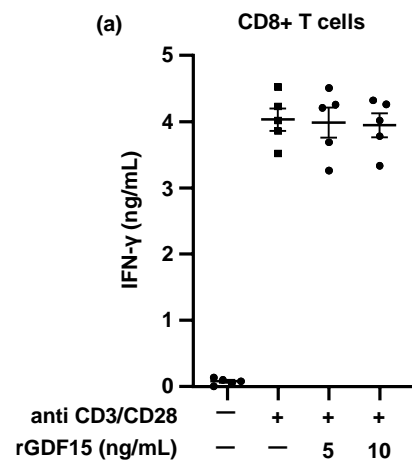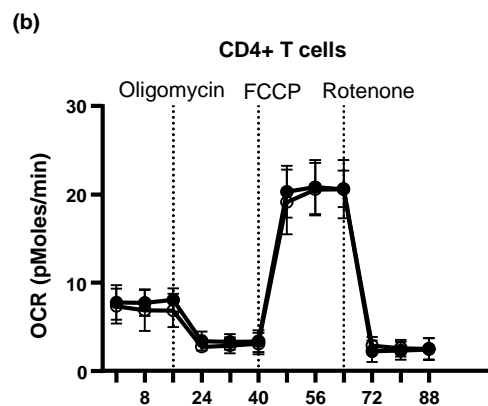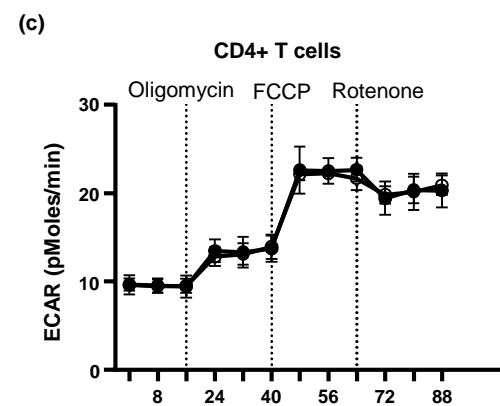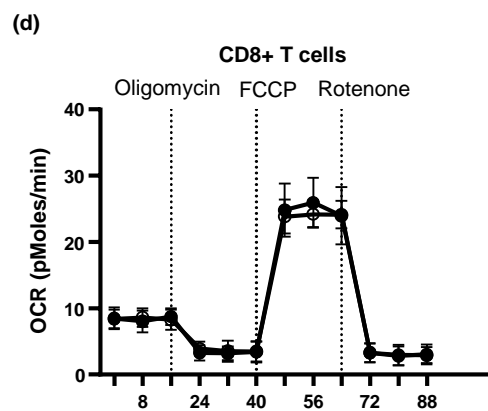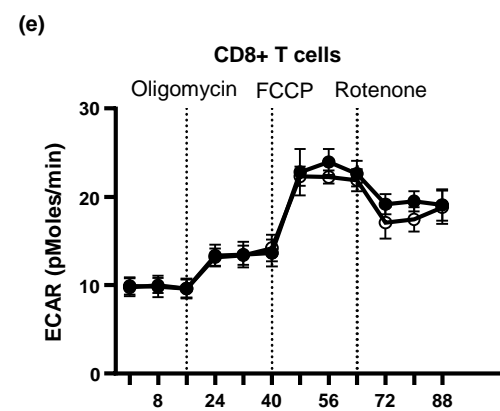

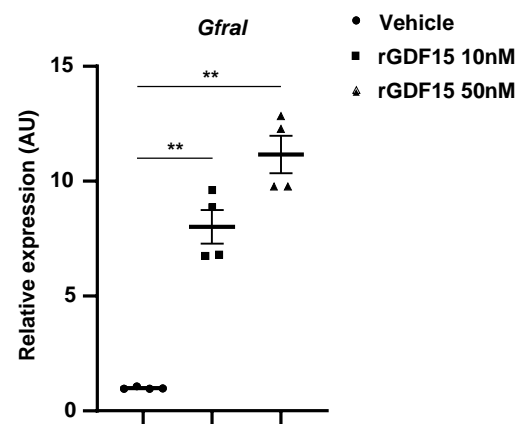

Supplement: Supplementary file 1 — Figure S1‐S13 [file ACEL-19-e13195-s001.pdf]
