# Supplementary material for: Growth differentiation factor 15 protects against the aging‐mediated systemic inflammatory response in humans and mice
Source: Aging Cell. 2020 Jul 21;19(8):e13195. doi: 10.1111/acel.13195 (PMC7431835; doi:10.1111/acel.13195)
Supplement: Supplementary file 2 — Table S1 [file ACEL-19-e13195-s002.docx]

**Supplemental Table 1:** **Demographics and baseline characteristics of human subjects**

| Variables | Control (*n* = 70) |
| --- | --- |
| Age, y | 52.5 ± 12.7 |
| Male, n (%) | 21 (30.0%) |
| BMI, kg/m^2^ | 23.8 ± 2.7 |
| FBG, mg/dL | 101.2 ± 13.6 |
| Fasting insulin, μIU/mL | 9.6 ± 4.9 |
| HOMA-IR | 2.4 ± 1.4 |
| HOMA-β-cell, % | 96.5 ± 51.7 |
| GDF15, mg/dL | 564.24 ± 140.4 |
| Total cholesterol, mg/dL | 202.2 ± 46.2 |
| Triglyceride, mg/dL | 117.0 ± 64.0 |
| HDL-C, mg/dL | 58.9 ± 15.1 |
| LDL-C, mg/dL | 119.0 ± 31.8 |
| Aspartate transaminase, IU/L | 23.3 ± 10.1 |
| Alanine transaminase, IU/L | 28.0 ± 21.1 |
| hsCRP, mg/dL | 1.0 ± 1.2 |

BMI, body mass index; FBG, fasting blood glucose; HOMA-IR, homeostasis model assessment-estimated insulin resistance; HOMA-β-cell, homeostasis model assessment-estimated beta cell; HDL-C, high-density lipoprotein cholesterol; LDL-C, low-density lipoprotein cholesterol; hsCRP, high-sensitivity C-reactive protein.
