# Supplementary material for: Growth differentiation factor 15 protects against the aging‐mediated systemic inflammatory response in humans and mice
Source: Aging Cell. 2020 Jul 21;19(8):e13195. doi: 10.1111/acel.13195 (PMC7431835; doi:10.1111/acel.13195)
Supplement: Supplementary file 7 — Table S6 [file ACEL-19-e13195-s007.docx]

**Supplemental Table 6: Primers used for real-time PCR**

| Genes | Forward (5’-3’) | Reverse (5’-3’) |
| --- | --- | --- |
| *Gdf15* | GAGCTACGGGGTCGCTTC | GGGACCCCAATCTCACCT |
| *GDF15* (human) | TGCGGAAACGCTAC AGGAC | GGAACAGAGCCCGGTGAAGG |
| *Gfral* | TTCCTGGCTGTTACGTTAAGC | GCCATTTGCATCAATCAAGCA |
| *Tnf* | AAGCCTGTAGCCCACGTCGTA | AAGGTACAACCCATCGGCTGG |
| *TNF* (human) | CCTCTCTCTAATCAGCCCTCTG | GAGGACCTGGGAGTAGATGAG |
| *Il1b* | GCCCATCCTCTGTGACTCAT | AGGCCACAGGTATTTTGTCG |
| *Il6* | TCCATCCAGTTGCCTTCTTG | TTCCACGATTTCCCAGAGAAC |
| *Ccl2* | TCAGCCAGATGCAGTTAACGC | TCTGGACCCATTCCTTCTTGG |
| *Cd11c* | CAAAATCTCCAACCCATGCT | CACCACCAGGGTCTTCAAGT |
| *Srebp1c* | ATCGCAAACAAGCTGACCTG | AGATCCAGGTTTGAGGTGGG |
| *Fasn* | CCCTTGATGAAGAGGGATCA | ACTCCACAGGTGGGAACAAG |
| *Col1a1* | TCCTCCAGGGATCCAACGA | GGCAGGCGGGAGGTCTT |
| *GADPH* (human) | CTGGGCTACACTGAGCACC | AAGTGGTCGTTGAGGGCAATG |
| *18s* | CTGGTTGATCCTGCCAGTAG | CGACCAAAGGAACCATAACT |
