# Supplementary material for: Growth differentiation factor 15 protects against the aging‐mediated systemic inflammatory response in humans and mice
Source: Aging Cell. 2020 Jul 21;19(8):e13195. doi: 10.1111/acel.13195 (PMC7431835; doi:10.1111/acel.13195)
Supplement: Supplementary file 8 — Supinfo [file ACEL-19-e13195-s008.docx]

**Growth differentiation factor 15 protects against the aging-mediated systemic inflammatory response in humans and mice**

- Ji Sun Moon^1#^, Ludger J.E. Goeminne^2#^, Jung Tae Kim^1,3#^, Jing Wen Tian^1,3^, Seok-Hwan Kim^4^, Ha Thi Nga^1,3^, Seul Gi Kang^1,3^, Baeki E. Kang^5^, Jin-Seok Byun^6^, Young-Sun Lee^7^, Jae-han Jeon^8^, Minho Shong^1,3^, Johan Auwerx^2^, Dongryeol Ryu^5,9,10^*, Hyon-Seung Yi^1,3^*

^1^Research Center for Endocrine and Metabolic Diseases, Chungnam National University Hospital, Chungnam National University School of Medicine, Daejeon 35015, Republic of Korea. ^2^Laboratory of Integrative Systems Physiology, École Polytechnique Fédérale de Lausanne (EPFL), CH-1015 Lausanne, Switzerland. ^3^Department of Medical Science, Chungnam National University School of Medicine, 266 Munhwaro, Daejeon 35015, Republic of Korea. ^4^Department of Surgery, Chungnam National University School of Medicine, Daejeon 35015, Republic of Korea. ^5^Department of Molecular Cell Biology, Sungkyunkwan University School of Medicine, Suwon, 16419, Republic of Korea. ^6^Department of Oral Medicine, School of Dentistry, Kyungpook National University, Daegu 41566, Republic of Korea. ^7^Department of Internal Medicine, Korea University College of Medicine, Seoul, 08308, Republic of Korea. ^8^Department of Internal Medicine, School of Medicine, Kyungpook National University, Daegu, Korea. ^9^Biomedical Institute for Convergence at SKKU (BICS), Sungkyunkwan University, Suwon, 16419, Republic of Korea. ^10^Samsung Biomedical Research Institute, Samsung Medical Center, Seoul, 06351, Republic of Korea.

- ^#^ These authors contributed equally to this work.

*Correspondence.

Hyon-Seung Yi, Research Center for Endocrine and Metabolic Diseases, Chungnam National University Hospital, Chungnam National University School of Medicine, Daejeon 35015, Republic of Korea **E-mail:** jmpbooks@cnu.ac.kr

- Dongryeol Ryu, Department of Molecular Cell Biology, Sungkyunkwan University School of Medicine, Suwon, 16419, Republic of Korea. **E-mail:** freefall@skku.edu

**Supplemental Experimental Procedures**

**Preparation of peripheral blood mononuclear cells (PBMCs)**

Samples of peripheral blood were obtained from the study participants. The blood samples were transferred into 10mL polystyrene tubes containing ethylenediaminetetraacetic acid (Sigma-Aldrich, Dorset, UK) as an anticoagulant. Then, PBMCs were isolated by centrifugation using a Ficoll-Hypaque density gradient (GE Healthcare Life Science, Buckinghamshire, UK) at room temperature. The PBMCs were tested to determine the number of viable cells in suspension and stained for flow cytometry analyses using directly conjugated monoclonal antibodies.

**Isolation and quantification of ccf-DNA and mtDNA levels in plasma from human subjects**

Plasma samples were separated in EDTA-coated collection tubes and stored in liquid nitrogen. The ccf-DNA was isolated from 100 μL of plasma using a Maxwell® RSC Instrument (AS4500, Promega, Madison, WI, USA) and a Maxwell® RSC ccf-DNA Plasma Kit (AS1480, Promega), according to the manufacturer’s instructions. The quantitation of dsDNA concentrations in ccf-DNA was assessed by fluorescent detection using a Quantus™ Fluorometer (E6150, Promega), according to the manufacture’s guidance. The concentrations of dsDNA were measured by QuantiFluor® dsDNA System (E2670, Promega). The abundance of circulating-cell free genomic (ccf nDNA) and mitochondrial DNA (ccf-mtDNA) was measured by quantitative real-time polymerase chain reaction (qPCR) (ABI Prism 7900HT Real-Time PCR System; Applied Biosystems, Foster City, CA, USA). Conditions for qPCR and sequences of the primers used for the amplification of mitochondrial gene encoding 16s-RNA (mtDNA79 and mtDNA230) and genomic DNA [Alu repetitive sequence 124 base pairs (bp) product] were previously described. Quantitation of internal standard plasmid was performed using M13 primers, as recommended by the manufacturer (Promega, Madison, WI, USA). Amplifications were performed using SYBR Green Master Mix (Life Technologies, Carlsbad, CA, USA), 1 µM forward/reverse primer, and 5 µl of ccf-DNA sample. The nDNA, mtDNA79, and mtDNA230 quantification cycle values were adjusted relative to the internal standard and calculated as relative expression using the 2^−ΔCt^ formula.

**Bioinformatics and statistical analysis using the BXD mouse reference population dataset**

Correlation analysis was performed using the datasets available from the GeneNetwork (http://www.genenetwork.org) for the BXD mouse genetic reference population. Kaplan-Meier estimates were used for analyzing the survival curves of BXD mice with higher (top 25%) or lower expression (bottom 25%) of *GDF15* transcripts. Metabolic phenotypes were also assessed in these BXD strains of mice fed with a normal chow diet or a high fat diet. All data analysis and plots were generated using R and RStudio (R Core Team and RStudio Team). The data were expressed as mean ± SEM, and p-values were calculated using a two-tailed Student’s t-test for pairwise comparisons, one-way ANOVA for multiple comparisons, and two-way ANOVA for multiple comparisons involving two independent variables.

**Isolation of hepatic mononuclear cells and adipose stromal vascular fraction**

Liver mononuclear cells were isolated, as previously described. Liver tissues from mice were cut into small pieces and incubated with pre-warmed media that included dissociation enzymes (Miltenyi Biotec, Bergisch Gladbach, Germany) for 30 min at 37°C. After enzymatic digestion, the hepatic cell suspensions were quickly homogenized in C-Tubes using the GentleMACS Dissociator (Miltenyi Biotec) and the m_liver_03 program. After debris removal, the cells were resuspended in phosphate-buffered saline and centrifuged at 1,000 × g for 5 min for hepatocyte elimination. The supernatants were removed by mechanical suction and filtered through a cell strainer with a 70 μm nylon filter (BD Falcon, Millville, NJ). Hepatic mononuclear cells were isolated by centrifugation at 1,200 × g for 10 min at 4°C and resuspended in RPMI-1640 medium (Welgene, Daegu, South Korea). Adipose tissues were minced into small pieces in pre-warmed RPMI-1640 media with type 1 collagenase. The minced tissues were digested with type 1 collagenase for 40 min at 37°C with gentle shaking and then filtered through a cell strainer with a 70 µm filter. Digested cells were collected by centrifugation at 800 × g for 5 min and incubated with RBC lysis buffer for 3 min. Then, these hepatic mononuclear cells and adipose stromal vascular fractions were used for analysis of the population and function of tissue-resident immune cells using flow cytometry staining (FACS) buffer.

**Real-time PCR analysis**

Real-time PCR analysis was performed using the primers listed in Table S6. Total RNA was extracted from the liver and adipose tissues using TRIzol Reagent (Invitrogen, Eugene, OR, USA), according to the manufacturer’s instructions. Complementary DNA (cDNA) was synthesized from the same quantity of RNA with M-MLV reverse transcriptase and oligo-dT primers (Invitrogen, Eugene, OR, USA) following the manufacturer’s instructions. Quantitative real-time PCR was performed using cDNA, 2× SYBR Green PCR Mix (Applied Biosystems, Foster City, CA, USA) and analyzed on an ABI Prism 7000 Sequence Detection System (Applied Biosystems, Foster City, CA, USA). The comparative Ct method was used to quantitate the transcripts, the expression of which was normalized to that of 18s RNA. The results were analyzed using the ΔΔCt method, and the values are expressed as fold differences relative to control.

**Staining**

Sections of the left and medial lobes of the liver or gonadal fat were fixed with 10% neutral buffered formalin (BBC Biochemical, Mt. Vernon, WA, USA). To examine histological features, sections (4 μm) of paraffin embedded liver tissue blocks were stained with 0.1% hematoxylin and eosin (Sigma-Aldrich). H&E staining was performed according to standard protocols. For immunohistochemistry, sections of gonadal fat were incubated with primary antibodies (anti-F4/80; 1:100; Abcam, Cambridge, UK) for 16 h at 4°C, and binding was detected using the Polink-1 HRP Rat-NM DAB Detection System (GBI Labs, Bothell, WA, USA).

Gonadal adipose tissues were collected from WT and *Gdf15* KO mice. The tissues were fixed in 1% paraformaldehyde for 1 hour and then washed with tap water. Samples were then incubated for 1 hour at room temperature in blocking solution containing 1% BSA in PBST (0.3% Triton X-100 in PBS). Next, the samples were incubated for 24 h at 4℃ with anti-CD11b (Abcam, Cambridge, MA, USA) and anti-F4/80 (Abcam, Cambridge, MA, USA) diluted 1:500 in PBS with 1% BSA. After washing, samples were incubated with a fluorescently labeled secondary antibody for 24 h at 4°C. Nuclei were stained using 4, 6-diamidino-2-phenylindole (DAPI; Sigma). After washing, samples were observed under a laser scanning confocal microscope (FV1000, Olympus Corp., Tokyo, Japan).

**Glucose tolerance and insulin tolerance test**

Twenty-month-old wild-type (WT) and *GDF15* KO mice were placed under fasting conditions for 16 hours prior to the glucose tolerance test. Next, 2 g of glucose per kilogram of body weight was injected into the intraperitoneal cavity, and blood glucose levels were measured using a glucometer (Accu-CHEK Active, Roche Diagnostics, IN, USA) at 0, 15, 30, 60, 90, and 120 min intervals. For the insulin tolerance test, the mice fasted for 6 hours prior to receiving an intraperitoneal injection of 0.75 U/kg insulin lispro (Humalog, Eli Lilly, Indianapolis, IN, USA), and levels of blood glucose were assessed at 15, 30, 60, 90, and 120 min intervals.

**Serum chemistry and ELISA**

Blood was collected from mouse hearts under general anesthesia. Blood was centrifuged at 10,000 rpm for 5 min, and serum was collected from supernatants. Biochemical measurement for ALT, AST, triglyceride, and total cholesterol was conducted using kits purchased from IDEXX Laboratories (ME, USA). Serum levels of TNF-α and IL-1β were measured by ELISA using kits obtained from the indicated supplier. Supernatants from the experiments of Th17 differentiation and CD4+ or CD8+ T cell activation with anti-CD3/CD28 were removed, and the levels of IL-17A and INF-γ were measured using a standard sandwich ELISA (BD biosciences, San Jose, CA).

**Measurement of hepatic triglyceride levels**

Using a mixture of chloroform and methanol (2:1 ratio), hepatic lipids were extracted from 100 mg liver tissue. Lipid extracts from liver tissues were lyophilized using nitrogen gas, and the triglyceride levels in the resuspended lipids were assessed by a Fuji Dri-Chem 4000i analyzer according to the manufacturer’s instructions (Fujifilm, Tokyo, Japan).

**Th17 differentiation**

Naïve CD4+ T cells were obtained from the PBMCs by negative selection using a human naïve CD4 isolation kit (Miltenyi Biotec Inc., Auburn, CA, USA) according to the manufacturer's instructions. 2.5 × 10^5^ naïve CD4+ T cells per well were seeded into 96-well plates, and then, the cells were stimulated with anti-CD3 (2 μg/mL)/CD28 (5 μg/mL), anti-INF-γ (10 μg/ml, R&D Systems), anti-IL-4 (10 μg/ml, R&D Systems), anti-IL-6 (50 μg/ml, R&D Systems), and anti-TGF-β (1 ng/ml; Cell Signaling Technology) for 5 days. The cells were incubated with ionomycin (500 ng/ml), PMA (5 ng/ml), and Golgi plug (1 μg/ml) for 5 h and harvested on day 5. The permeabilized cells were washed and resuspended in 1% formaldehyde and further stained for intracellular cytokines with anti-CD4-AF700 and anti-IL-17A-APC. Multicolor flow cytometry was performed using a LSR Fortessa (BD Biosciences, San Jose, CA, USA), and the data were analyzed using FlowJo software (Tree Star, Ashland, OR, USA).

**Regulatory T cell differentiation**

Naïve CD4+ T cells were obtained from spleen and lymph nodes of WT mice. The naïve CD4+ T cells were treated with anti-B220, CD11b, CD11c, CD19, CD24, CD25, CD44, CD8 antibodies (Biolegend, San Diego, CA, USA) at concentrations of 1μg per 10^7^ cells. Then, they were combined with streptavidin-negative selection beads (Invitrogen, Eugene, OR, USA) and negatively selected using a MagniSortTM magnet (Invitrogen, Eugene, OR, USA). The cells were cultured (5 x 10^5^ / well) in 24-well plates coated with anti-CD3e (2.5μg/ml; Invitrogen, Eugene, OR, USA) antibodies, and anti-CD28 (4μg/ml. BD Biosciences, San Jose, CA, USA) antibodies, recombinant mouse IL-2 protein (20ng/ml; R&D Systems, Minneapolis, MN, USA), recombinant mouse TGF-β protein (5ng/ml; R&D Systems, Minneapolis, MN, USA), 2-Mercaptoethanol (55μM; Invitrogen, Eugene, OR, USA), and 10% fetal bovine serum in RPMI 1,640 medium for 4 days. Treg induction was measured by flow cytometry by analyzing Foxp3 expression in CD25+CD4+ T cells.

**Measurement of oxygen consumption rate and extracellular acidification rate**

Mitochondrial oxygen consumption rate (OCR) and extracellular acidification rate (ECAR) were measured using a Seahorse XF-96 Extracellular Flux Analyzer (Seahorse Bioscience Inc., North Billerica, MA, USA). CD4+ or CD8+ T cells were sorted using a magnetic CD4+ or CD8+ T cell isolation kit (Miltenyi Biotec Inc., Auburn, CA, USA). CD4+ or CD8+ T cells were cultured and activated by anti-CD3 (2 μg/mL)/CD28 (5 μg/mL) in Seahorse XF-96 plates at a density of 50,000 cells per well. Next, the cells were incubated with RPMI-1640 medium lacking sodium bicarbonate at 37°C in a non-CO_2_-containing incubator for 1 h. The medium and mitochondrial OxPhos inhibitors were adjusted to pH 7.4 on the day of the OCR assay. Measurements of OCR and ECAR were assessed under basal conditions and after adding 1 µM oligomycin, 0.5 µM FCCP, and 0.5 µM rotenone. OCR and ECAR were calculated and recorded by a sensor cartridge and Seahorse XF-96 software.

**Legends to supplemental figures**

**Figure S1. Association of GDF15 with inflammation markers in young and elderly subjects.** (a,b) Serum levels of GDF15 and hepatic *Gdf15* expression in young (8-week-old; n = 6) and old (20-month-old; n = 6) mice. (c) Hepatic expression of *TNF* in young and elderly subjects. (c) Violin plot visualizing *Gdf15* expression in various organs of WT 21-month-old mice using RNA-seq database from Tabula Muris Senis. (d) Violin plot visualizing Hepatic *Gdf15* expression from WT 1-month-old mice to 27-month-old mice using RNA-seq database from Tabula Muris Senis. (e) Hepatic *TNF* expression in young (≤ 40; n = 8) and elderly (≥ 60; n = 8) subjects. (f) Hepatic *TNF* expression in subjects with serum GDF15 in the bottom (n = 8; mean age, 40.8 years old) or top (n = 8; mean age, 60.5 years old) 25% groups. (g-j) Characterization of the absolute number of naïve and memory T cell subsets in the PBMCs from young (≤ 40-year-old; n = 14) and elderly (≥ 60-year-old; n = 24) subjects. (k) Correlation analysis of serum GDF15 levels and memory CD8+ T cells of peripheral blood from human subjects. (l-o) Production of Granzyme B in senescent CD4+ and CD8+ T cells of PBMCs from young (≤ 40-year-old; n = 14) and elderly (≥ 60-year-old; n = 24) subjects. Data are expressed as mean ± SEM. **P* < 0.05, ***P* < 0.01 ((a,b,e,f,i,j,n,o): two-tailed t-tests, (k): simple linear regression).

**Figure S2. Levels of serum GDF15 skeletal muscle- or adipocyte-specific Crif1-deficient mice and correlation between serum GDF15 levels and ccf-mtDNA copy number in human subjects.** (a) Correlation analysis of serum GDF15 levels and ccf-mtDNA copy number of peripheral blood from human subjects. **(b)** Serum levels of GDF15 in controls and skeletal muscle- (n = 5) or adipocyte-specific *Crif1*-deficient mice at 8 weeks of age (n = 5) (MKO and AdKO, respectively). Data are expressed as mean ± SEM. **P < 0.01 (a): simple linear regression, (b): two-tailed t-tests).

**Figure S3. Volcano plots of differential gene expression analysis between the top 25% and the bottom 25% groups according to *GDF15* levels.** Differentially expressed genes are represented as red dots. The blue dot indicates the location of *GDF15*. The FDR threshold of 5% is marked as a black line

**Figure S4. Analysis of related pathways regulated by *GDF15* expression in the muscle dataset from the GTEx database.** (a) Distribution of 804 skeletal muscle *GDF15* expression levels (log_2_(TPM+0.001)) for human subjects in GTEx . The red and blue boxes represent the top 25% (n = 201) and the bottom 25% (n = 201) groups according to *GDF15* levels, respectively. (b) The number of DEGs between the top 25% and the bottom 25% groups according to *GDF15* levels. The red, blue, and gray represent the number of up-regulated, down-regulated, and un-regulated genes, respectively. (c) KEGG pathway analysis of the DEA results. The red, blue, and gray boxes indicate up-regulated, down-regulated, and un-regulated pathways, respectively. Bar plots representing the up-regulated (red) and down-regulated (blue) pathways for significantly enriched pathways. The pathways shown in this bar plot were selected from the significant pathways (FDR < 0.1) in the KEGG analysis. (d) Skeletal muscle *GDF15* expression correlates positively with *AP1, TNFAIP3, NOD2, IFNG, CCL2, CD44, CD11B,* and *CD3E* gene expression. The correlation analysis was conducted by GEPIA2 in the GTEx skeletal muscle dataset (R: Pearson’s correlation coefficient).

**Figure S5. Gene Set Enrichment Analysis of mitochondrial stress and translation in the liver of** **BXD mice.** (a) The diagram shows the result of Gene Set Enrichment Analysis of mitochondrial stress and translation, including the enrichment scores in the BXD mice with higher and lower hepatic *Gdf15* expression. (b) Heatmap analysis based on the genes related to mitochondrial stress and quality control between the BXD mice with higher and lower hepatic *Gdf15* expression.

**Figure S6. Role of GDF15 on metabolic phenotypes and cold resistance in mouse BXD reference populations.** (a–e) Food intake over a 24-h period (a), body weight (b), fat mass (c), lean mass (d), and liver mass (e) of the *Gdf15*-low (blue) and *Gdf15*-Hi (red) groups under a normal chow diet (n = 3–4 per each of 20 BXD lines). (f–j) Food intake over a 24-h period (f), body weight (g), fat mass (h), lean mass (i), liver mass (j) of the *Gdf15*-low (blue) and *Gdf15*-Hi (red) groups under a high fat diet for 21 weeks (n = 3–4 per each 20 BXD lines). (k, l) Body temperature during acute cold exposure at 4℃ was measured in the *Gdf15*-low (blue) and *Gdf15*-Hi (red) groups under a normal chow diet (k) or a high fat diet (l) for 21 weeks (n = 3–4 per each 20 BXD lines). (m) Plasma MCP1 level of the *Gdf15*-low (blue) and *Gdf15*-Hi (red) group under a high fat diet for 21 weeks (n = 3–4 per each 20 BXD lines). Data are expressed as mean ± SEM. **P* < 0.05, ****P* < 0.001 (two-tailed t-tests).

**Figure S7. Gating strategy for the analysis of CD4+, CD8+ T cells, monocytes, and neutrophils in liver.**

**Figure S8. Analysis of hepatic infiltrating immune cells of 8-week-old or 20-month-old WT and *Gdf15* KO mice.** (a) Absolute number of hepatic mononuclear cells of 20-month-old WT (n = 6) and *GDF15* KO (n = 6) mice. (b) Population of NK, NKT, CD4+ T and CD8+ T cells in the livers of 20-month-old WT and *GDF15* KO mice. (c,d) Representative flow cytometry plots of T, natural killer, and natural killer T cells in liver tissues of WT (n = 6) or *Gdf15* KO (n = 6) mice. (e,f) Population size and frequency of CD44+CD62L- and CD44-CD62L- in CD4+, and CD8+ T cells in liver tissues of WT (n = 6) or *Gdf15* KO (n = 6) mice. (g–i) Population size and frequency of CD44+CD62L- and CD44-CD62L- of CD4+ or CD8+ T cells in the liver of 8-week-old WT and *GDF15* KO mice. (j,k) Representative FACS plot and statistical analysis of hepatic monocytes and neutrophils in 20-month-old WT and *GDF15* KO mice. (l–p) Representative FACS plot of IFN-γ, TNF-α or IL-17A producing CD4+ and CD8+ T cells in liver tissues of WT (n = 6) or *Gdf15* KO (n = 6) mice. Data are expressed as mean ± SEM. *P < 0.05, ***P* < 0.01 ((a,b,i,k): two-tailed t-tests).

**Figure S9. FACS Analysis of immune cells from mesenteric lymph nodes of 20-month-old WT and *Gdf15* KO mice.** (a) Population sizes and frequencies of NK cells and CD3+ T cells in the mesenteric lymph nodes of 20-month-old WT and *Gdf15* KO mice. (b–e) Population of CD44+CD62L- and CD44-CD62L- of CD4+ or CD8+ T cells in the liver of 20-month-old WT and *Gdf15* KO mice. Data are expressed as mean ± SEM (two-tailed t-tests).

**Figure S10. FACS Analysis of adipose immune cells of 20-month-old WT and *Gdf15* KO mice.** (a,b) Population of CD4+ T and CD8+ T cells in the gonadal adipose tissues of 20-month-old WT (n = 6) or *Gdf15* KO (n = 6) mice. (c) Population size and frequency of CD44+CD62L- and CD44-CD62L- in CD4+, and CD8+ T cells in the gonadal fat tissues of WT (n = 6) or *Gdf15* KO (n = 6) mice. (d–f) Representative FACS plots of macrophages, monocytes and neutrophils in the gonadal adipose tissues of WT (n = 6) or *Gdf15* KO (n = 6) mice. Data are expressed as mean ± SEM. ***P* < 0.01 ((b,c): two-tailed t-tests).

**Figure S11. Analysis of the metabolic phenotype in 20-month-old WT and *Gdf15* KO mice.** (a) Measurement of triglyceride levels in the liver of 20-month-old WT and *Gdf15* KO mice. (b) Representative images of adipose CD11b+/F4/80+ macrophages from young and old WT and *Gdf15* KO mice (scale bar = 10 μm). (c) Percentage of F4/80 positive cells within the gonadal adipose tissues of 20-month-old WT and *Gdf15* KO mice. (d) Transcripts of genes related to adipogenesis and inflammatory cytokines in the gonadal adipose tissues of 20-month-old WT and *Gdf15* KO mice. Data are expressed as mean ± SEM. **P* < 0.05, ***P* < 0.01 ((a,c,d): two-tailed t-tests).

**Figure S12. Role of GDF15 in metabolic switching of the CD4+ and CD8+ T cells.** (a) IFN-γ production from differentiated CD8+ T cells stimulated with anti-CD3 (2 μg/mL)/CD28 (5 μg/mL) in the presence or absence of the indicated concentrations of recombinant GDF15. (b–e) Oxygen consumption rate and extracellular acidification rate (ECAR), measured in CD4+ or CD8+ T cells treated with or without recombinant GDF15. Data are expressed as mean ± SEM ((a): one-way ANOVA, (b-e): two-tailed t-tests).

**Figure S13. Effect of recombinant GDF15 on *Gfral* expression during Treg differentiation.** Data are expressed as mean ± SEM. **P < 0.01 (one-way ANOVA)
